# An Overview of Reviews on the Association of Low Calorie Sweetener Consumption With Body Weight and Adiposity

**DOI:** 10.1016/j.advnut.2024.100239

**Published:** 2024-08-08

**Authors:** Kelly A Higgins, Rita Rawal, Matthew Kramer, David J Baer, Aaron Yerke, David M Klurfeld

**Affiliations:** 1 United States Department of Agriculture (USDA), Agricultural Research Service, Beltsville Human Nutrition Research Center, Beltsville, MD, United States; 2 Exponent Inc., Chemical Regulatory & Food Safety, Washington, DC, United States; 3 United States Department of Agriculture (USDA), Agricultural Research Service, United States; 4 Indiana University School of Public Health -Bloomington, Bloomington, IN, United States

**Keywords:** nonnutritive sweeteners, sweetening agents, adiposity, body composition, umbrella review, humans

## Abstract

**Background:**

Numerous systematic reviews (SR) and meta-analyses (MA) on low calorie sweeteners (LCS) have been published in recent years, concluding that LCS have beneficial, neutral, or detrimental effects on various health outcomes, depending on the review.

**Objectives:**

The objective of this overview of reviews was to determine how the methodologies of SR investigating the association between LCS consumption and body weight (BW) influence their findings and whether MA results can provide a consistent estimated effect.

**Methods:**

Systematic searches of PubMed, Scopus, and Cochrane Library were conducted in November 2022 to identify SR of randomized controlled trials (RCT) or non-randomized studies (NRS) investigating the association between LCS consumption and BW. The methods, MA results, and conclusions were extracted from each eligible SR.

**Results:**

Of the 985 search results, 20 SR evaluated the association between LCS and BW, drawing from publications of 75 RCT, 42 prospective cohort studies, and 10 cross-sectional studies. There was a considerable lack of overlap of studies included within each SR attributed, in part, to the inclusion of studies based on design; thus, each SR synthesized results from distinctly different studies. Evidence synthesis methods were heterogeneous and often opaque, making it difficult to determine why results from certain studies were excluded or why disparate results were observed between SR.

**Conclusions:**

SR investigating the effect of LCS on BW implement different methodologies to answer allegedly the same question, drawing from a different set of heterogeneous studies, ignoring the basic assumptions required for MA, resulting in disparate results and conclusions. Previous MA show the large effects of study design, which results in inconsistent estimates of the effect of LCS on BW between MA of RCT and NRS. Given the availability of long-term RCT, these studies should be the basis of determining causal relationships (or lack thereof) between LCS and BW.

This trial was registered at PROSPERO as CRD42022351200.

**Statement of Significance**

Previously published systematic reviews and meta-analyses that attempt to answer the question “what is the estimated effect of low calorie sweetener consumption on body weight?” report disparate results and conclusions because these reviews attempt to synthesize findings from studies that were designed to test specific but different research questions.

## Introduction

There is consensus among the nutrition community that individuals should limit their intake of added sugars, owing to the association between added sugars intake and risk of obesity, type 2 diabetes mellitus (T2D), cardiovascular disease, and dental caries [[1], [2], [3], [4], [5]]. There is no consensus as to what added sugars should be replaced with, if replaced at all. Low calorie sweeteners (LCS) can be used as substitutes for added sugars, maintaining sweetness without energy. Although LCS found in the food supply are regulated food additives that are generally recognized as safe by the US Food and Drug Administration, the Joint FAO/WHO Expert Committee on Food Additives, and the European Food Safety Authority, their use in the food supply is contentious, particularly as it relates to the effect of (or lack thereof) LCS on body weight (BW).

Dietary guidance should be based on established causal relationships determined through the conduct of high-quality randomized controlled trials (RCT), in which the treatment and control are randomized to account for observed confounding factors, which may affect the relationship between exposure X and outcome Y. This is especially important when investigating the relationship between LCS intake and BW, because LCS are often consumed to manage BW or induce weight loss. Given that long-term (i.e., ≥1 y) RCT designed to investigate the effect of LCS consumption on BW are possible [[6], [7], [8], [9], [10]], high-quality experimental evidence should be the basis for drawing conclusions. However, recent dietary guidance from the WHO recommends reduced intake of added sugars while avoiding LCS [7], which was based on results from meta-analyses (MA) of nonrandomized studies (NRS), not results from MA of RCT, which were also conducted as part of the review process [11]. This is concerning, because reviews that rely on NRS are biased toward concluding adverse effects of LCS on BW whereas RCT are more likely to conclude beneficial or null effects [12,13].

Systematic reviews (SR) and MA are increasingly popular tools to perform evidence synthesis of RCT or NRS to develop medical and dietary guidance [2], including dietary guidance regarding the use of LCS. MA and meta-regression are attractive tools for evidence synthesis, because they statistically combine multiple studies to address broad questions [14]. MA and meta-regression, like other statistical methods, requires satisfying assumptions to produce meaningful and unbiased results. If all primary studies included within an MA measure the same effect and are a representative sample of all studies conducted addressing a specific question, then the resulting MA should provide a better estimate of the true effect size than any single primary study and estimated study-to-study variability is due to a random error [14]. In the best-case scenario, the included studies are so similar that they could be considered blocks of a larger study; where the subjects provide an unbiased representation of the general or target population, the independent and dependent variables are identically defined and measured, the experimental design and duration of each study is the same, there are no complicating factors that affect one study but not another, and there are a sufficient number of included studies (there is no agreed upon minimum number to yield meaningful results, but a minimum of 2 studies is required) [15,16]. When these assumptions are not met and studies included within an MA are highly heterogeneous, inclusion or exclusion of individual studies can lead to large deviations in effect estimates, making it difficult to interpret the estimate effect of dietary exposure X on health outcome Y. This may explain why SR and MA designed to address the same question of the effect of a dietary exposure on a health outcome often come to different conclusions, some of which can be implausible and/or misleading [21,22], sparking debates as to whether SR and MA should be the basis to draw scientific conclusions [[23], [24], [25], [26]], particularly conclusions based on data collected from nutrition studies [21,22,27,28].

Numerous SR and MA on LCS have been published in recent years, concluding that LCS are beneficial, detrimental, or have no effect on various health outcomes, including BW and adiposity. The objective of this overview of reviews (OoR) was to determine how the inclusion criteria, methodologies, and availability of relevant data used in SR influence the findings and whether MA results can provide a consistent estimated effect among SR investigating the association of LCS consumption with BW-related outcomes.

## Methods

### Overview

An OoR (also referred to as an umbrella review) was conducted to systematically search and identify multiple relevant SR on a topic area for the purpose of extracting and analyzing the results [17]. The protocol for this OoR was registered on PROSPERO (CRD42022351200) *a priori* and followed the recommendations from the Cochrane Handbook [9]. The Population, Intervention/Exposure, Comparator, Outcome, and Study Design or Setting [PI(E)COS] criteria to identify SR eligible for inclusion are summarized in Table 1. A complete list of the inclusion/exclusion criteria is summarized in Supplemental Methods.

**TABLE 1:** PI(E)COS criteria to determine inclusion in the overview of reviews.

Population	Generally healthy populations or with a disease prevalent among Western populations (e.g., obesity, hypertension, hyperlipidemia, T2D, and metabolic syndrome) No restriction based on age
Intervention/exposure	Intake of any type of LCS used alone or in combination with other foods, beverages, LCS, or energetic sweeteners No restriction on intervention/exposure duration
Comparators	No restrictions on comparators, including lower dose of LCS, no LCS, energetic sweeteners, no intervention, placebo, or comparison with other LCS
Outcomes	Measures of adiposity, including BW, BMI, prevalence of overweight/obesity, fat and lean body mass, WC, and/or W:H ratio effect estimates
Study design/setting	Systematic reviews of RCT Systematic reviews of NRS

Abbreviations: BW, body weight; LCS, low calorie sweetener; NRS, non-randomized study; PI(E)COS, Population, Intervention/Exposure, Comparator, Outcome, and Study Design or Setting; RCT, randomized controlled trial; T2D, type 2 diabetes mellitus; WC, waist circumference; W:H, waist-to-hip ratio.

### Literature search

Literature searches were conducted through PubMed, Scopus, and Cochrane Library in November 2022. There were no restrictions on the publication period. Research presented in languages other than English was excluded. The search strings were peer reviewed by 2 external reviewers using Canadian Agency for Drugs and Technologies in Health 2015 Peer Review of Electronic Search Strategies before running the search [29]. In addition, a list of 12 publications expected to be recovered in the literature search was identified by the external reviewers [11,19,[30], [31], [32], [33], [34], [35], [36], [37], [38], [39]] and was used to pilot the search strings. The search strings used and the number of publications recovered from each search are provided in Supplemental Methods.

### Screening and selection

Screening of the search results was managed using Covidence systematic review software (Veritas Health Innovation). Two reviewers (KAH, RR) independently determined whether a publication met the inclusion/exclusion criteria based on the title and abstract. Publications that both reviewers determined to be ineligible during the first round of screening were excluded. During the second round of screening, 2 reviewers (KAH, RR) independently determined whether the publications met the inclusion/exclusion criteria based on the entire article with discrepancies resolved by a third reviewer (DJB, DMK) if necessary. OoR or scoping reviews recovered in the literature search were identified; a search of the reference list was conducted to ensure that all relevant SR identified in the OoRs were included in the current OoR. A PRISMA flow diagram was completed to outline the identification of SR for the current OoR [40].

### Data extraction

Study characteristics were extracted from eligible SR and MA independently by 2 reviewers with differences reconciled by a third reviewer. For all identified SR and MA, the following information was extracted: review type, aim of the review, PI(E)COS inclusion/exclusion criteria, literature search methods, evidence synthesis (including MA) methods, risk of bias (ROB) assessment methods, study quality assessment methods, individual studies included within the review, MA results for BW-related outcomes, certainty of evidence [i.e., Grading of Recommendations, Assessment, Development, and Evaluations (GRADE)], conclusions, and funding source (see Supplemental Methods for additional details on the data extracted). The mean difference (MD), standardized mean difference (SMD), and corresponding CI from the primary MA and specific subgroup analyses [e.g., MA by specific PI(E)COS] were extracted from each SR along with measures of interstudy heterogeneity (referred to herein as heterogeneity) and statistical significance of the effect estimate. Published SR protocols (if available) were referenced for relevant information. Missing information in the SR and MA was noted if not available; SR authors were not contacted, and the primary studies included in the SR and MA were not referenced.

### Risk of bias and study quality

A Measurement Tool to Assess Systematic Reviews (AMSTAR 2) was used to evaluate ROB and study quality of the included SR [41]. Two reviewers independently critically appraised each of the identified SR (KAH, DMK), with discrepancies resolved by a third reviewer (DJB).

### Statistical analysis

Per the preregistered protocol (CRD42022351200), we planned to statistically analyze the MA results reported in the included SR using multiple regression and/or partial least squares regression to determine what study design factors influenced the observed BW-related outcome effect estimates. The goal was to use the study and population characteristics and MD and/or SMD values for each of the included studies as reported in the most recent MA to conduct the analysis, based on the assumption that these MA would be the most comprehensive and included most if not all the relevant primary studies. These MA were determined to be the MA by Rios-Leyvraz and Montez [11], which investigated health effects associated with LCS interventions/exposures from RCT and NRS regardless of the comparator and when compared with sugars and water, and the MA by Rogers and Appleton [36], which investigated LCS intake compared with sugar, water, or nothing, with and without oral exposure to sweetness (i.e., LCS capsules compared with placebo capsules) in RCT. The key study and population characteristics investigated were characteristics commonly reported in SR and characteristics hypothesized to introduce interstudy heterogeneity, including but were not limited to study design, study country, population age, population sex, population BW status, population health status, LCS administered, LCS dose, LCS vehicle, comparator, duration, whether the study was a weight loss intervention, LCS consumption status at baseline, ROB, and funding source.

However, it became apparent that synthesis of the RCT data from these 2 MA was not appropriate. First, MD values were reported in 1 article [11], whereas SMD values converted to relevant units were reported in the other [33]. Although it is possible to convert MD to SMD values (and vice versa), it could not be accomplished given the information provided in each of the MA. Second, in 1 MA [11], treatment and/or comparator arms within the same study were combined to allow the “best estimate of the effect of” LCS; whereas in the other MA [36], each treatment or comparator group was treated as a separate study but they did not account for dependencies owing to a shared control group [42]. Third, 1 MA analyzed parallel arm and crossover studies separately to account for reduced within-study variance among crossover studies [36], whereas the other MA combined results from parallel arm and crossover studies but performed a sensitivity analysis excluding crossover studies [11]. Finally, 1 MA conducted separate analyses for end BW/BMI values and change BW/BMI values [36], whereas the other MA extracted only change from baseline values [11]. Owing to these differences in the analysis and reporting of results, we determined that primary studies needed to be examined to extract results from each study in a consistent way. This was out of scope of the current OoR, and thus, a statistical analysis using only MA results was not formally conducted.

### Data synthesis

All figures, tables, and the accompanying database were created using Microsoft Excel and R (version 4.3.1). The database of extracted data and code used to create the figures is available on OSF (osf.io/bnj7w) [43].

The individual studies included within each SR were tabulated and the overlap of studies included in the different SR were depicted with a Sankey plot using networkD3 (version 0.4). Owing to the lack of overlap of studies included with each SR identified in this OoR, efforts were made to determine the cause of these discrepancies, including investigating differences in the aim, PI(E)COS criteria, literature search strategy, and evidence synthesis methods between SR. The primary aim and the corresponding PI(E)COS criteria for each of the identified SR was used to separate the SR into categories; specifically, categories based on study design, primary intervention/exposure, and population age. Each SR was classified by included study design: RCT only, NRS only, or both RCT and NRS. The primary intervention/exposure investigated was classified as any LCS, low calorie sweetened beverage (LCSB) only, specific LCS, or other. Owing to the differences in BW change between children or adolescents and adults independent of LCS intake, SR that included studies conducted exclusively among children and/or adolescents were evaluated separately from SR among adults or all age groups. The PI(E)COS criteria of each of the included studies were aggregated by the primary intervention/exposure of the SR to display differences in the inclusion/exclusion criteria across the included SR.

Next, the results from the MA conducted within each SR were compared to determine whether these differences in study inclusion resulted in differences in overall effect estimates. MD and SMD values were aggregated by study design (RCT, prospective cohort, and cross-sectional), outcome [BW (in kilograms), BMI (in kg/m^2^), BMI z-score, body fat mass (in percentages and kilograms), body lean mass (in kilograms), waist circumference (WC), waist-to-hip ratio (W:H), hazard ratio (HR) of obesity, risk ratio (RR) of obesity, RR of overweight orobesity (OW/OB), odds ratio (OR) of OW/OB], and comparator [all comparators, sugars/ sugar -sweetened beverage (SSB), water/nothing/placebo for RCT and all comparators, sugars/SSB, lowest LCS/LCSB intake level, and lower LCS/LCSB for prospective cohort studies]. The MD, SMD, and corresponding CI from BW, BMI, and OR/HR of obesity from each of the primary MA and subanalyses were graphed in a forest plot to display the range of results from each of the MA. The forest plots were produced using ggplot2 (version 3.4.4). Only BW, BMI, and OR/HR of obesity results from MA conducted among adults were included in the forest plots owing to the limited number of studies conducted among children/adolescents and analyses of other measures of BW status and adiposity; results from MA among the total population were included if results among adults only were not available: all analyses [30]; select analyses [32,34]. Results from MA of studies among children/adolescents, all age groups combined, and specific subpopulations (e.g., populations with type 1 diabetes mellitus/T2D [[44], [45], [46]]; populations stratified by BW status [11,32,39], sex [34,47,48], age [34,37,47], country [47], and baseline LCSB/SSB consumption status [11]) are included in the OSF database. Select MA investigated interventions/exposure on specific sweeteners [11,38,45,46] or LCS delivered as a capsule without oral exposure to sweetness [36]; these results are included in the OSF database. When only 1 study was identified for a specific comparison, results were extracted but not investigated further.

Heterogeneity of results from the MA were characterized based on the following classification system: *I*^2^ values between 0% and 30%, heterogeneity might not be important; >30% and 50%, moderate heterogeneity; >50% and 75%, substantial heterogeneity; and >75% and 100%, considerable heterogeneity [18].

Conclusions drawn by each of the SR based on evidence from RCT and NRS (both prospective cohort studies and cross-sectional studies) were categorized as more beneficial (i.e., decrease BW), less beneficial (i.e., increase BW), or neutral (i.e., no directional effect or association), or evidence was insufficient to draw a conclusion; this classification scheme was adapted from a previous citation network analysis [13]. This information was used to chart the number of primary studies (both RCT and NRS) by the conclusions drawn by each of the SR using ggplot2.

The goal of this OoR was not to draw conclusions about the strength of reported associations; therefore, separate analyses by study quality or ROB were not conducted and certainty of evidence regarding the relationship between LCS and BW status was not evaluated.

## Results

### Overview of included SR

**FIGURE 1:**
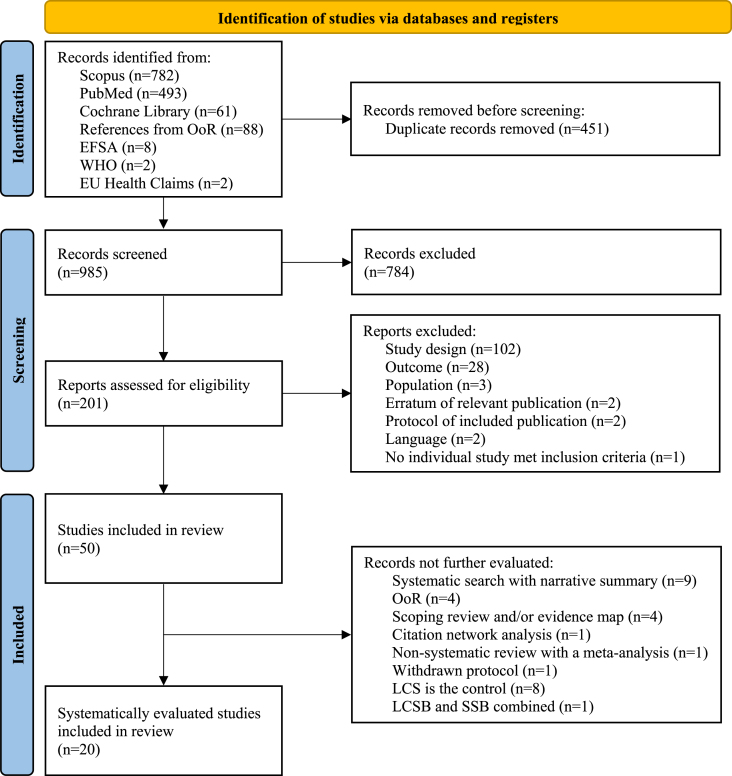
PRISMA flow diagram. BW, body weight; EFSA, European Food Safety Authority; EU, European Union; LCS, low calorie sweetener; LCSB, low calorie sweetened beverage; *n*, number of publications; OoR, overview of reviews; SSB, sugar-sweetened beverage.

The PRISMA flow diagram is presented in Figure 1. A total of 985 publications (duplicates removed) were identified in the literature search. During the title and abstract screen, 784 were excluded because they did not meet the inclusion/exclusion criteria; an additional 151 publications were excluded based on the full-text review (Supplemental Table 1). A total of 50 publications met the *a priori* inclusion criteria, but 30 of these publications were not further evaluated in the current OoR (rationale for exclusion provided in Figure 1 and Supplemental Table 2). Therefore, information from 20 SR that investigated the association between LCS and a BW-related outcomes were evaluated in this OoR [11,19,20,[30], [31], [32],[34], [35], [36], [37], [38], [39],[44], [45], [46], [47], [48],[49], [50], [51]]. One SR [11] was an update of a previously conducted SR and MA [39]; however, owing to differences between the 2 protocols, both SR were included in this OoR. One SR reported results from studies among adults and adolescents [30] in a separate publication from results from studies among children [35]; both SR were included in this OoR. The AMSTAR 2 assessment for each of the included SR is included in Table 2.

**TABLE 2:**
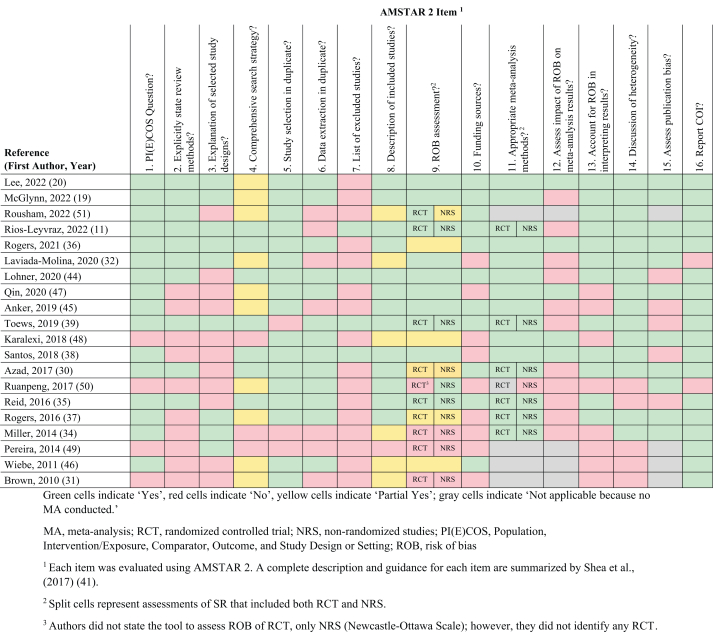
AMSTAR 2 assessment of included systematic reviews.

### Studies included within SR

**FIGURE 2:**
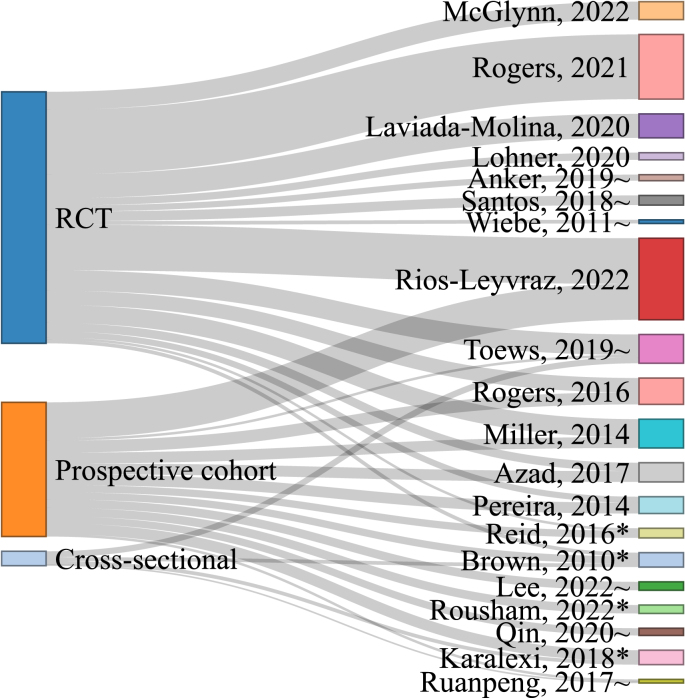
Sankey plot representing portion of studies included within each systemic review (SR). ∗SR that included children/adolescents only; ∼SR that included only adults; all other SR included all age groups. RCT, randomized controlled trial. An interactive figure can be found at https://osf.io/trwak. An interactive figure that indicates which individual study is included in each SR can be found here: https://osf.io/74cdu.

The studies included within each SR are represented in Figure 2; online interactive versions of the figure presenting which individual study is included in which SR are available on OSF (osf.io/bnj7w) [43]. The SR cumulatively identified 75 publications from RCT, 42 publications from prospective cohort studies, and 10 publications from cross-sectional studies. The number of publications summarizing results from prospective cohort studies included in the SR that included prospective cohorts ranged from 2 to 30; the number of publications of RCT or clinical trial (CT) included in SR that included RCT or CT ranged from 2 to 54. Cross-sectional studies were listed as included studies in 4 SR [11,31,39,50]. In addition to differences in studies that were identified within each SR, there were also differences in the number of studies included within each MA. The number of RCT included within an MA ranged from 1 (i.e., results from the primary research study, not an MA, given the 2-study requirement to mathematically synthesize results) to 29 studies whereas the number of prospective cohort studies included with an MA ranged from 1 to 9 studies. In some cases, specific studies were summarized in text only and not included within the MA because the primary studies did not report sufficient data or reported data in a format that could not be easily incorporated into an MA.

### Primary aim and PI(E)COS

The primary aim of each of the included SR varied, which influenced the PI(E)COS criteria adopted for each SR (summarized in Table 3). The interventions/exposures of interest included any or all LCS [11,[30], [31], [32],[34], [35], [36], [37],^39^,^44^,^48^]; LCSB and/or SSB only [19,20,47,49,50]; specific LCS (i.e., steviol glycosides, aspartame) [38,45]; “unhealthy foods and beverages” (defined by the authors as foods and beverages that were high in LCS) [51]; and sweetener additives (including LCS) [46]. The primary outcome of interest was BW-related outcomes in 7 SR [32,34,36,37,[49], [50], [51]], whereas 13 SR aimed to evaluate a variety of health outcomes, including BW [11,19,20,30,31,35,38,39,[44], [45], [46], [47], [48]]. Most reviews included studies with energetic and nonenergetic, sweet and nonsweet comparators; no or lower intake of the intervention/exposure; or reported limited or no inclusion/exclusion criteria related to the comparator.

**TABLE 3:** PI(E)COS criteria of included systemic reviews^1^.

Reference^2^	Population^3^	Intervention/exposure^4^	Comparator	Study Design^5^	Duration
***Intervention/Exposure:Any LCS***					
Rios-Leyvraz and Montez, 2022 [11]	Generally healthy Adults (≥18 y)^6^ and children (<18 y) All BW statuses	Inclusion: LCS, by name or not, alone or in combination with other LCS; consumed at levels within ADI^7^ Exclusion: Sugar alcohols and natural caloric sweeteners; LCS intake explicitly exceeded the ADI	Inclusion: No or lower dose of LCS, including comparison with any type of sugar, placebo, plain water, or no intervention. Concomitant interventions included if concomitant interventions were similar in both arms Exclusion: Comparison with other LCS	Inclusion: RCT (parallel, crossover, cluster), non-randomized CT^11^, prospective cohort, case–control, cross-sectional Exclusion: case reports, case series, ecologic studies, reviews, SRMA	≥7 d
Rogers et al., 2021 [36]	All health statuses All ages All BW statuses	Inclusion: Any LCS, regardless of mode of delivery, including the use of instructions to consume LCS, to continue consuming food/beverage containing LCS, or to consume capsules containing LCS	Inclusion: Equivalent food/beverage without LCS (food/beverage containing sugar, unsweetened food/beverage); no additional food/beverage (usual diet, waitlist control); or placebo (presumably inert) capsules. Concomitant interventions included if concomitant interventions were similar in both arms. Exclusion: LCS treatment confounded with another treatment not controlled for in comparator	Inclusion: RCT (parallel, crossover)	≥1 wk
Laviada-Molina et al., 2020 [32]	Generally healthy All ages All BW statuses	Inclusion: Aspartame, saccharin, sucralose, stevia, cyclamate, and acesulfame-K	Inclusion: Energetic (sucrose, high fructose corn syrup) and nonenergetic comparators (water, placebo, or nothing)	Inclusion: RCT	≥4 wk
Lohner et al., 2020 [44]	T1D, T2D Age NS BW status NS	Inclusion: Any LCS consumed alone or in combination with another LCS; studies that provided LCS as part of a behavior-changing intervention	Inclusion: Usual diet, no intervention, placebo, water, different LCS, different dose of same LCS, energetic sweetener. Concomitant interventions included if concomitant interventions were similar in both arms	Inclusion: RCT	≥4 wk
Toews et al., 2019 [39]	Generally healthy Adults (≥18 y) and children (<18 y) All BW statuses	Inclusion: Any LCS, either alone or in combination with other LCS, within the ADI or did not report any information on dose; studies that applied concomitant interventions were included as long as the interventions were similar and equally balanced between the intervention and comparator groups to establish fair comparisons; LCS foods, beverage, or packets Exclusion: Type of LCS not reported; LCS intake explicitly exceeded the ADI	Inclusion: Any alternative intervention, including any other type of energetic or nonenergetic sweetener, any type of sugar, no intervention, placebo, or plain water	Inclusion: RCT (parallel, crossover, cluster), nonrandomized CT, prospective cohort, retrospective cohort, case–control, cross-sectional	≥7 d
Karalexi et al., 2018 [48]	Health status NS Children and adolescents BW status NS	Inclusion: Any LCS items	Inclusion: NS	Inclusion: Prospective cohort, case–control, cross-sectional^8^ Exclusion: RCT, case reports, case series, in vitro studies, animal studies, reviews	NS
Azad et al., 2017 [30]	Health status NS Adults and adolescents (>12 y) BW status NS	Inclusion: Any LCS Exclusion: Analysis of LCS effects was not fully prospective (associations presented for change in LCS intake but not for baseline intakes)	Inclusion: Placebo, lower intake of LCS, or no LCS Exclusion: LCS effects cannot be examined independently of other intervention components. LCS associations with outcomes of interest were not adjusted for confounders/covariates	Inclusion: RCT (parallel), prospective cohort Exclusion: RCT (quasi-, crossover, cluster), cross-sectional, retrospective cohort, case–control, reviews, commentaries	≥6 mo
Reid et al., 2016 [35]	Health status NS Infants and children (<12 y)^6^ BW status NS	Inclusion: All LCS consumed as ingredients or additives to food/beverage Exclusion: Analysis of LCS effects was not fully prospective (associations presented for change in LCS intake but not for baseline intakes)	Inclusion: Energetic sweetener, placebo, or usual diet Exclusion: LCS effects cannot be examined independently of other intervention components	Inclusion: RCT, prospective cohort Exclusion: RCT (quasi-, crossover, cluster), cross-sectional, retrospective cohort, case–control, reviews, commentaries	≥6 mo
Rogers et al., 2016 [37]	Generally healthy Age NS All BW statuses	RCT Inclusion: LCSB or LCS sweetened foods Prospective cohort inclusion: LCSB or LCS	Inclusion: NS	Inclusion: RCT (parallel, crossover), prospective cohort Exclusion: Abstract only	RCT, ≥4 wk Prospective cohort, >1 y follow-up
Miller and Perez, 2014 [34]	Generally healthy Adults and children BW status NS	Inclusion: Intake of ≥1 LCS (including sugar alcohols)	Inclusion: Control (not further specified); could be examined independent of control arm	Inclusion: RCT, prospective cohort	RCT, ≥2 wk Prospective cohort, ≥6 mo
Brown et al., 2010 [31]	Health status NS Children and adolescents (≤18 y) BW status NS	Inclusion: Any LCS	Inclusion: NS	Inclusion: RCT, prospective cohort, cross-sectional	NS
***Intervention/Exposure:LCSB***					
Lee et al., 2022 [20]	Generally healthy Adults >19 y BW status NS	Inclusion: LCSBs, defined as “diet” or “low-calorie and no-calorie” beverage where energetic sugars are replaced with no-energy (e.g., aspartame and sucralose) and/or low energy (e.g., stevia) sweeteners	Inclusion: SSB or water	Inclusion: prospective cohort	≥1 y
McGlynn et al., 2022 [19]	All health statuses Adults BW status NS	Inclusion: SSB, LCSB, or water alone or while on a weight-loss or nutrition education program Exclusion*:* Multimodal interventions; nonusual intake method (LCS capsules) LCS added to foods, fortified beverage or nutrient-dense beverage (milk and juice)^9^	Inclusion: SSB, LCSB, or water Exclusion: Did not use comparator groups containing ≥1 of the other beverage interventions	Inclusion: RCT	≥2 wk
Qin et al., 2020 [47]	Health status NS Adults (≥18 y) BW status NS	Inclusion: LCSB or SSB (not further specified)	NS	Inclusion: prospective cohort	NS
Ruanpeng et al., 2017 [50]	Health status NS Adults BW status NS	Inclusion: LCSB or SSB (not further specified)	Inclusion: No intake of soda	Inclusion: RCT, prospective cohort, case–control, cross-sectional	NS
Pereira, 2014 [49]	Health status NS Adults and youth BW status NS	Inclusion: LCSB or SSB (not further specified) Exclusion: Combination of LCSB and SSB as 1 exposure variable	Inclusion: NS	Inclusion: RCT, prospective cohort	RCT, NS Prospective cohort, ≥6 mo
***Intervention/Exposure:Specific LCS***					
Anker et al., 2019 [45]	Generally healthy, T1D, T2D, hypertension Adults ≥18 y BW status NS	Inclusion: Orally administered steviol glycosides Exclusion: Steviol glycosides combined with other dietary supplements	Inclusion: Placebo matching the intervention	Inclusion: RCT	No restriction
Santos et al., 2018 [38]	Health status NS Adults BW status NS	Inclusion: Aspartame	Inclusion: No consumption of aspartame	Inclusion: RCT Exclusion: Reviews, letters, personal opinions, book chapters, and conference abstracts	NS
***Intervention/Exposure:Other***					
Rousham et al., 2022 [51]	Generally healthy Children <10.9 y BW status NS	Inclusion: Consumption of unhealthy food/beverage, including food/beverage high in LCS Exclusion: Not consumption of unhealthy food/beverage Studies reporting only dietary patterns or eating practices	Inclusion: Less unhealthy foods and beverage (no or low LCS)	Inclusion: RCT, nonrandomized CT, pre/post with a control, prospective cohort, retrospective cohort Exclusion: CT without a control, cross-sectional, case–control, narrative reviews, SRMA	NS
Wiebe et al., 2011 [46]	Generally healthy, T1D, T2D Adults (≥16 y) All BW statuses	Inclusion: Sweetener additives including LCS aspartame, saccharin, stevioside, or sucralose	Inclusion: Any sweetener (nonenergetic, sugar alcohols, and saccharides) Exclusion: Placebo controls^10^	Inclusion: RCT (parallel, crossover)	≥1 wk

Abbreviations: ADI, acceptable daily intake; CT, clinical trial; LCS, low calorie sweetener; LCSB, low calorie sweetened beverage; MA, meta-analysis; NS, not specified; RCT, randomized controlled trial; SR, systematic review.

1 References are sorted by the LCS exposure (any LCS, LCSB only, specific LCS, and other) and publication year. Study outcomes are not listed in this table, because all reviews included BW-related outcomes as a primary or secondary outcome. Specific BW-related outcomes and additional health outcomes included within each review are included in the supplemental database (www.osf.io/bnj7w) [43].

2 First author and publication year.

3 Sex is not an inclusion criterion for any of the reviews. Specific age cutoffs not specified unless otherwise listed.

4 Inclusion/exclusion criteria related to LCS vehicle is listed; if vehicle criterion is not listed, criterion was not specified in the included review.

5 RCT were assumed to include both crossover and parallel unless specifically reported.

6 SR also included studies among pregnant women; however, these results were not summarized in the current OoR because of the *a priori* criteria to exclude SR among pregnant women.

7 Included if unclear whether an ADI had been exceeded.

8 Although not explicitly stated, cross-sectional studies were included in this SR and MA.

9 Due to the presence of other macronutrients.

10 In order to investigate comparative effectiveness of different sweeteners, as opposed to exploring the implications of avoiding sweeteners altogether.

11 Non-randomized CT listed as both an inclusion and exclusion criteria in this study. However, non-randomized CT were included in the list of included studies within this review.

Inclusion criteria based on study design of included studies varied across the identified SR. Of the 20 SR, 7 included only RCT [19,32,36,38,[44], [45], [46]]; 3 included only prospective cohorts and/or case–control studies [20,47,48]; and 10 included RCT and NRS (including prospective cohorts, retrospective cohorts, case–control, cross-sectional, and non-randomized clinical trials) [11,30,31,34,35,37,39,[49], [50], [51]].

A specific population of interest based on health status or age was the focus of some of the included SR. The primary aim of 4 SR was to investigate the effect of LCS consumption exclusively among infants, children, or adolescents [31,35,48,51], whereas 7 SR focused exclusively among adults [19,20,38,[45], [46], [47],^50^]. Seven SR restricted to studies among generally healthy populations [11,20,32,34,37,39,51], whereas 1 SR included exclusively populations with type 1 diabetes mellitus or T2D [44].

### Literature search methods

Differences in literature search strategy were observed between the SR (summarized in the OSF database), including differences in the source of publications (number of databases or databases searched), language limits, and search terms. Search terms varied in whether only general terms for LCS were used (e.g., LCS, high intensity sweeteners, and artificial sweeteners); whether terms for specific LCS (e.g., aspartame, sucralose, and saccharin) or other food/diet-related terms (e.g., diet drinks, reduced sugar, and low calorie) were included; whether BW-related terms or other health-related terms were included; or whether study design terms were included.

### Evidence synthesis methods

The evidence synthesis methods used in each SR are reported in the OSF database. Most of the included SR conducted MA (*n* = 15) or network MA (*n* = 1). Two SR established *a priori* methods to conduct an MA but determined there were an insufficient number of studies to do so [46,51], and 2 SR were designed to only conduct qualitative analysis (i.e., no MA) [31,49]. Many SR used multiple analysis methods to combine the results from the included studies. Among the SR that completed quantitative evidence synthesis, meta-analytical methods included random-effects MA [11,19,20,30,32,[34], [35], [36], [37], [38], [39],^44^,^45^,^47^,^48^,^50^], meta-regression [11,20,32,36,37,47], fixed-effects MA [20,34,36,37,39], dose-response MA [20,47], and random-effects network MA [19].

Subgroup analyses and/or sensitivity analyses were conducted in 12 SR [11,19,20,32,34,36,37,39,44,45,47,48]; 2 SR planned to conducted subgroup analyses *a priori* but did not conduct them owing to insufficient number of studies [30,35]. The most common subgroup analyses were based on comparator [11,19,20,32,34,36,37,44], age [11,32,34,37,39,47], ROB or study quality [11,34,36,47,48], sex [32,34,47,48], and BW status [11,32,39].

### MA results

**FIGURE 3:**
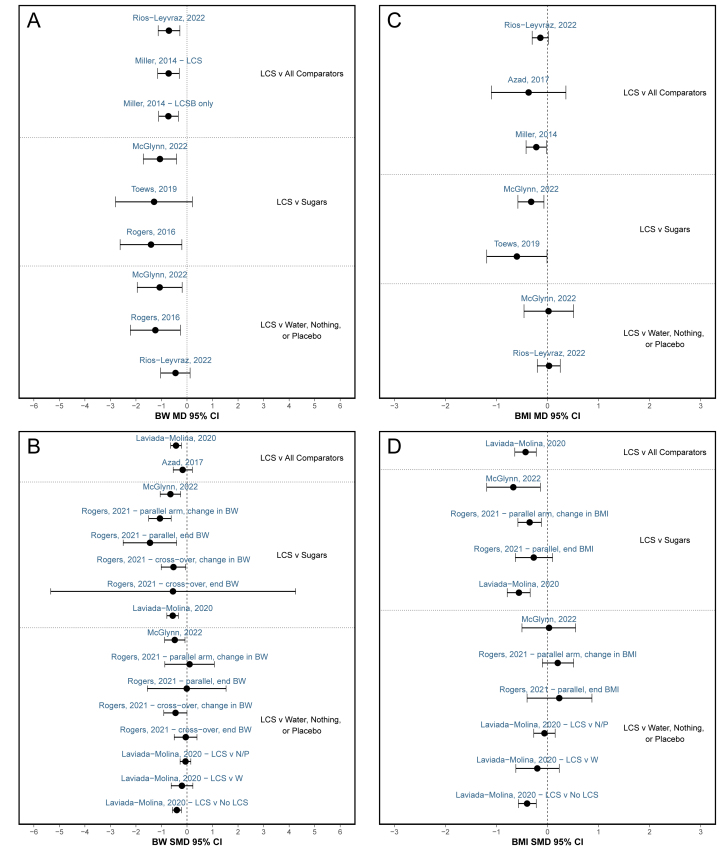
Mean difference (MD) and standardized mean difference (SMD) in body weight (BW) and BMI results from meta-analyses of RCT. Meta-analysis results investigating LCS vs. various comparators on BW and BMI from RCT among adults. Each panel displays results of (**A**) MD in BW; (**B**) SMD in BW; (**C**) MD in BMI; and (**D**) SMD in BMI. The effect estimates within the same block represent similar comparators, listed to the side. Results from meta-analyses of studies with specific LCS, LCS delivered as a capsule, and among children and specific subpopulations are included in the OSF database (osf.io/bnj7w) [43]. References listed as author last name and year above MD or SMD effect estimate. Additional information (e.g., RCT design type, result values included, and comparator) provided if multiple relevant meta-analyses were conducted in the same SR. BW, body weight; LCS, low calorie sweetener; LCSB, low calorie sweetened beverage; MD, mean difference; N, no intervention/nothing; P, placebo; SMD, standardized mean difference; v, versus; W, water.

**FIGURE 4:**
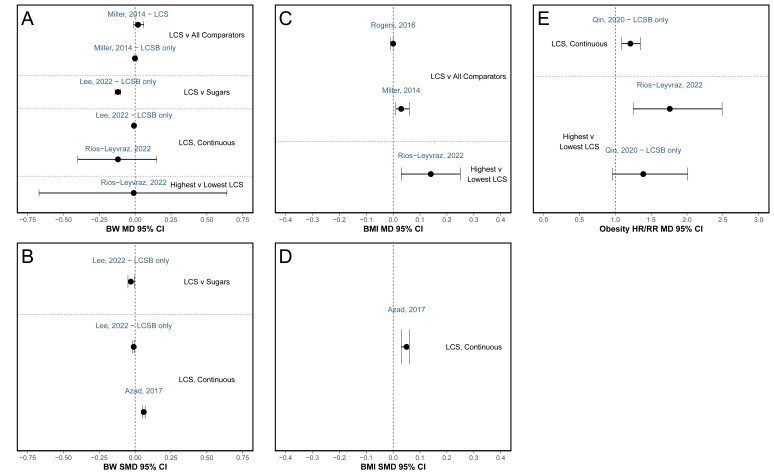
Mean difference (MD) and standardized mean difference (SMD) in body weight (BW), BMI, and obesity risk results from meta-analyses of cohort studies. Meta-analysis results investigating LCS vs. various comparators on BW, BMI, and obesity HR or RR from cohort studies among the adults. Each panel displays results of (**A**) MD in BW; (**B**) SMD in BW; (**C**) MD in BMI; (**D**) SMD in BMI; (**E**) MD in obesity HR or RR. The effect estimates within the same block represent similar comparators, listed to the side. Results from meta-analyses of studies among children and specific subpopulations are included in the OSF database (osf.io/bnj7w) [43]. References listed as author last name and year above MD or SMD effect estimate. Additional information (e.g., exposure type) provided if multiple relevant meta-analyses were conducted in the same SR. BW, body weight; HR, hazard ratio; LCS, low calorie sweetener; LCSB, low calorie sweetened beverage; MD, mean difference; RR, risk ratio; SMD, standardized mean difference; v, versus.

The MD and SMD results from the MA of the effect of LCS on BW and BMI among adults from each of the SR are depicted in FIGURE 3, FIGURE 4 and the OSF database. Among MA that included data from RCT conducted among adults, the reported MD in BW between LCS interventions compared with any comparator ranged from −1.41 kg (95% CI: −2.62, −0.20 kg) to −0.45 kg (95% CI: −1.03, 0.12; *n* = 9 MA) (Figure 3A) and the reported SMD in BW ranged from −1.45 kg (95% CI: −2.5, −0.41 kg) to 0.10 kg (95% CI: −0.87, 1.07 kg; *n* = 16) (Figure 3B). Among all MA of RCT, MD or SMD was statistically significant in favor of LCS or were not statistically significant; no results were statistically significantly in favor of the comparator. Therefore, despite differences in the SR methodology and differences in the magnitude of the effect estimate, results from MA of RCT consistently yielded the same direction of effect. Results varied slightly when specific comparators were investigated as delineated in FIGURE 3, FIGURE 4. Overall MD and SMD values were greater when LCS were compared with sugars or SSB than when compared with water, nothing, or a placebo. Most of the MA reported substantial heterogeneity (*I*^2^ ≥ 50%; *n* = 23 of the 37 MA that reported heterogeneity). Among the MA with relatively low heterogeneity (*I*^2^ ≤ 30%, *n* = 12), the median number of studies included in the MA was 8 studies; thus, caution should be used when using *I*^2^ for assessing the degree of heterogeneity given substantial uncertainty of *I*^2^ estimates with small study counts [18].

Conversely, among MA that included data from prospective cohort studies conducted among adults, MD in BW ranged from −0.12 kg (95% CI: −0.4, 0.15 kg) to 0.02 kg (95% CI: −0.01, 0.06 kg; *n* = 6) (Figure 4A); MD in BMI ranged from 0.00 (95% CI: −0.01, 0.00) to 0.14 (95% CI: 0.03, 0.25; *n* = 3) (Figure 4C); and MD when presented as HR or RR of obesity ranged from 1.21 (95% CI: 1.09, 1.35) to 1.76 (95% CI: 1.25, 2.49; *n* = 3) (Figure 4E) when LCS intake was compared with any comparator. These MA results of prospective cohort studies among adults were statistically significant in favor of LCS [20], in favor of the control (i.e., no or low LCS) [11,30,34,47], or were not significant [11,34,37,47]. Only 1 substitutional analysis compared LCSB intake with SSB among prospective cohort studies, concluding substitution of LCSB for SSB was associated with significantly lower BW (MD: −0.12 kg/y; 95% CI: −0.14, −0.10 kg/y) [20].

### SR conclusions

**FIGURE 5:**
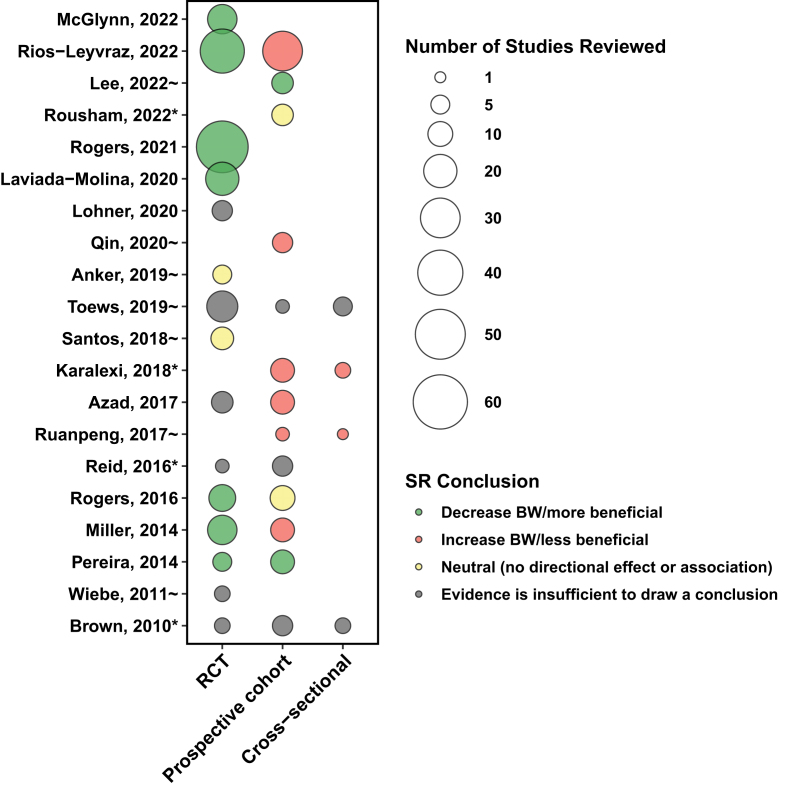
Number of primary RCT and NRS included within a SR by SR conclusion of the association between LCS and BW. ∗SR that included children/adolescents only; ∼SR that included only adults; all other SR included all age groups. Conclusions drawn by each of the SR based on evidence from RCT or NRS and the number of RCT, cohort studies, and cross-sectional studies reviewed. The conclusion classification scheme was adapted from a previous citation network analysis [40]. SR sorted in a reverse chronologic order.

The conclusions drawn by each of the SR depended on the type of studies included in the review (Figure 5). Based on review of evidence from RCT, 9 SR concluded LCS can have a beneficial impact [11,19,32,34,36,37,49] or neutral effect on BW [38,45], whereas 6 SR considered evidence to be insufficient to draw conclusions [30,31,35,39,44,46]. When NRS were considered, 4 of the SR concluded LCS have a beneficial impact or neutral effect on BW [20,37,49,51], whereas 6 SR concluded that LCS have an adverse effect on BW [11,30,34,47,48,50]. Some of the conclusions depended on the comparator; specifically, LCS were beneficial when compared with sugars [11,19,20,32,36,37,49].

Certainty of evidence was evaluated systematically using GRADE and/or NutriGRADE in 8 of the identified SR [11,19,20,32,38,39,44,51] (see Supplemental Table 3 and the OSF database). The certainty of evidence ranged from very low to moderate. Despite recommendations from Cochrane to downgrade evidence due to ROB inherent to NRS (i.e., confounding and selection bias) [52], ROB was considered to be not serious for most assessments of results from NRS [11,20]. Directness was considered not serious with the exception of 2 SR [20,32], despite differences in the population, intervention/exposure, and comparators of the included studies. Given the range of GRADE determinations between reviews designed to address the same question, these determinations should be interpreted with caution.

## Discussion

Systematic reviews designed to address the association between LCS consumption with BW-related outcomes use different inclusion criteria, literature search methods, and evidence synthesis methods that result in the combination of highly heterogeneous studies each designed to address their own specific question. Despite these differences in methodology, there was a consistent pattern that MA of results from RCT suggest that LCS are beneficial for BW management (particularly when compared with sugars) or have no effect on BW. This is highlighted with the results from 2 recent reviews with different inclusion criteria, evidence synthesis methods, and reporting of results [11,36]. Conversely, BW status effect estimates of MA of NRS produce a range of results from significantly beneficial to significantly adverse. Although ≤29 RCT could be combined within 1 MA [11], it was only possible to include 9 cohort studies within an MA [37], which is likely too few studies to yield statistically reliable, unbiased results given the heterogeneity of the included studies [14] despite the precise effect estimates from MA of NRS [53,54]. The disparate conclusions drawn from the SR and MA is predominantly due to whether RCT were included and the basis for conclusions.

Fundamentally differences between RCT and NRS contribute to differences in effect estimates observed in the included SR and MA. RCT are considered the highest quality evidence owing to the randomization of the treatment, reducing the impact of confounding; however, RCT are often limited by their short duration and external validity. Alternatively, prospective cohort studies capture information from larger populations for longer periods, making it possible to track long-term adverse effects [54]. Yet, causality cannot be determined because of unobserved confounding or biasing factors that influence the relationship between the exposure and outcome, including self-selection of the exposure: specific to this review, selecting to consume LCS in response to weight gain (i.e., reverse causality). RCT and NRS also tend to explore different interventions/exposures and comparators. RCT can be biased by noncompliance, whereas NRS are hindered by exposure measurement error, which is often infrequent and self-reported [28,55]. Specific to LCS, the NRS investigating the association between LCS and BW almost exclusively evaluate frequency of LCSB intake (not total LCS intake) compared with lower frequency of intake or no intake, which provide little to no information about whether LCSB serve as a substitute for other energetic alternatives, such as SSB. This has important implications for BW yet was only explored in 1 SR of prospective cohort studies [20]. The limitations of individual studies, particularly among NRS, become inflated when combined in an MA, producing “very precise but equally spurious results,” which should be interpreted with caution or not conducted at all [53,54].

One could argue that MA are an inappropriate tool for evaluating the relationship between LCS and BW. Each study that is included in an SR or MA was designed to address its own specific research questions and used unique methods to do so. The SR examined in this OoR combined results from studies among diverse populations and settings; which test the effect of many different LCS or combinations of LCS, which include distinct chemicals with different chemical structures, taste profiles, receptor affinity in the oral cavity and throughout the gastrointestinal tract, digestive and metabolic fates, and effects on BW [[56], [57], [58], [59], [60]]; delivered alone or in combination with other sweeteners; in a variety of vehicles; and for a range of durations. These LCS are compared with a variety of controls that vary widely in sweetness and energy content, with and without concomitant interventions. Set of primary studies, failing to meet the basic assumptions necessary to yield a meaningful estimate of the effect of LCS on BW. When the available studies within an SR are so heterogeneous and do not measure the same effect, one should not be asking simply if there is a treatment effect on means (or variances) but rather what factors are responsible for the heterogeneity of study results. Subgroup analyses (conducted in 12 of the 16 SR that completed an MA) are helpful for determining sources of heterogeneity and can be more informative and reliable despite combining results from a fewer number of primary studies; for example, factoring in study duration [11,36] or investigating change and substitution effects [20]. Ignoring high heterogeneity among studies included in an MA is a mistake and not only introduces confusion but also can result in erroneous conclusions.

This OoR is not without its own limitations. Although the bulk of the literature was likely captured in the literature search, select publications published after the search (November 2022) may have been missed. This OoR attempts to synthesize results from SR, which have numerous limitations. Data were compiled as reported within each review; reanalysis of the data from the identified MA was not conducted. Data extracted from each review were limited to the criteria established *a priori*. A systematic evaluation of SR quality was conducted as part of this OoR but did not capture all concerns. AMSTAR 2 provides a critical appraisal of the quality of the methods but includes limited evaluation of the integrity of the analysis methods, only using 1 question to determine when appropriate methods were used for the statistical combination of results. Data extraction and statistical errors are very common in SR and MA [[61], [62], [63]]; yet, common errors are not evaluated as part of review assessment tools. Although attempts were made to highlight major errors in the included SR and MA, it was not in the scope of this article to detail every error. The goal was not to draw conclusions about the strength of reported associations; therefore, certainty of evidence was not graded and no conclusions regarding the relationship between LCS and BW status were drawn.

In conclusion, SR investigating the effect of LCS on BW implement different methodologies to answer similar, but different, research questions, pooling highly heterogeneous primary research. Thus, basic assumptions of MA were not satisfied, making their results questionable. The number of primary studies identified in each SR varied widely. Despite these differences, results were generally consistent, in which MA of results from RCT indicate LCS are beneficial for BW management, particularly when compared with sugars, whereas SR that included results from NRS tend to be adverse or null. More SR are not going to lead the field any closer to answering whether there is a universal causal relationship (or lack thereof) between LCS and BW. Instead of trying to statistically combine studies to explain the complex relationship between LCS intake and BW, using potentially inappropriate statistical methods for this question that continually yield mixed results, it is more appropriate to rely on the high-quality RCT designed to test specific hypotheses and mechanisms to explain under which conditions LCS intake decreases, increases, or has no effect on BW and develop dietary recommendations for LCS.

## Acknowledgments

A special thank you to Katherine M. Appleton, PhD (Bournemouth University) and Marissa M. Shams-White (National Cancer Institute, National Institutes of Health) for completing the search string peer review.

## Author contributions

The authors’ responsibilities were as follows – KAH, DJB, MK, DMK: designed the research; KAH, RR, DJB, MK, DMK: conducted the research; KAH, MK: analyzed data and performed statistical analyses; KAH, AY: produced the figures; KAH: wrote the initial draft, with edits made by other authors; KAH: had primary responsibility for final content; and all authors: have read and approved the final manuscript.

### Conflict of interest

KAH is an employee at Exponent from August 2023 to present and was from January 2019 to April 2021; received funds for research from USDA-ARS, USDA-NIFA, and Hass Avocado Board; serves as President to the Institute of Food Technologists (IFT) Washington, DC section; and has a close relative employed by a consumer-packaged goods company. DJB serves as federal liaison to the Institute for the Advancement of Food and Nutrition Sciences (IAFNS) Lipids and Carbohydrates committees; received funds from USDA-ARS, California Walnut Commission, Qualisoy, Almond Board of California, American Pulse Association, USDA-NIFA, NIH/NIDCD, National Multiple Sclerosis Society, National Cattlemen’s Beef Association, Department of Defense, and Hass Avocado Board. DMK has served as a consultant to the National Cattleman’s Beef Association, Potatoes USA, the Calorie Control Council, Dyets, General Mills, the University of Arkansas for Medical Sciences, and USDA-ARS; is an Associate Editor of the *Journal of Nutrition*; and is an elected member of the IAFNS Board of Trustees. RR, MK, and AY have no conflicts or funds to disclose.

### Funding

Salaries for KAH, RR, AY, and DJB were covered by the USDA’s congressional salary allocation. DMK received no compensation for his work on the project. MK received an honorarium from the Institute for the Advancement of Food and Nutrition Sciences (IAFNS) Low- and No-Calorie Sweeteners Committee (IAFNS-KRAMER-202200914). IAFNS is a nonprofit science organization that pools funding from industry and advances science through the in-kind and financial contributions from private and public sector members. IAFNS had no involvement in the design, analysis, interpretation, or presentation of the data and results.

### Data availability

Data described in the manuscript are made publicly and freely available without restriction at https://osf.io/bnj7w/.

## Appendix A Supplementary data

Multimedia component1 Multimedia component1
