# Supplementary material for: An Overview of Reviews on the Association of Low Calorie Sweetener Consumption With Body Weight and Adiposity
Source: Adv Nutr. 2024 Aug 8;15(12):100239. doi: 10.1016/j.advnut.2024.100239 (PMC11705604; doi:10.1016/j.advnut.2024.100239)
Supplement: Multimedia component1 [file mmc1.docx]

**Supplemental Materials**

Table of Contents

[**Supplemental Methods** 2](#_Toc165803357)

[Search Strings 2](#_Toc165803358)

[Supplemental searches in gray literature 7](#_Toc165803359)

[PI(E)COS Criteria 8](#_Toc165803360)

[Data Extraction Template 11](#_Toc165803361)

[**Supplemental Tables** 23](#_Toc165803362)

[Supplemental Table 1. List of excluded studies based on full text review 23](#_Toc165803363)

[Supplemental Table 2. List of publications that met the a priori inclusion criteria but were not evaluated further in the current overview or reviews 40](#_Toc165803364)

[Supplemental Table 3. Certainty of evidence of the association between LCS intake and BW-related outcomes as reported in select systematic reviews 44](#_Toc165803365)

# **Supplemental Methods**

## Search Strings

| **Database** | **Search String** | **Filters** | **Results ^1^** |
| --- | --- | --- | --- |
| **PubMed** | (sweet*[tiab] OR "non-nutritive sweeteners"[mesh] OR acetosulfame[Supplementary Concept] OR acesulf*[tiab] OR acetosulfam*[tiab] OR "ace K"[tiab] OR ace-K[tiab] OR advantame[tiab] OR alitame[tiab] OR aspartame[mesh] OR aspartam*[tiab] OR aspartylphenylalanine[tiab] OR brazzein*[tiab] OR canderel[tiab] OR cyclamate*[tiab] OR cyclamates[mesh] OR "cyclamic acid"[tiab] OR Enliten[tiab] OR "Equal"[tiab] OR Goldswite[tiab] OR grosvenorii[tiab] OR hermesetas[tiab] OR "Luo Han Guo"[tiab] OR milisucre[tiab] OR mogroside*[tiab] OR monellin[tiab] OR "monk fruit"[tiab] OR neohesperidin[tiab] OR neotame[tiab] OR Nectresse[tiab] OR Nozucar[tiab] OR Newtame[tiab] OR nutrasweet[tiab] OR phyllodulcin[tiab] OR PureLo[tiab] OR "Pure Lo"[tiab] OR PureVia[tiab] OR "Pure Via"[tiab] OR rebaudioside*[tiab] OR rebuadiana[tiab] OR rebiana[tiab] OR rebaudianum[tiab] OR rebaudianums[tiab] OR saccharin[mesh] OR saccharin*[tiab] OR "Siraitia grosvenorii"[tiab] OR splenda[tiab] OR stevia[tiab] OR stevias[tiab] OR stevia[mesh] OR steviol*[tiab] OR stevioside*[tiab] OR sucralose[tiab] OR "Sugar Twin"[tiab] OR Sunett[tiab] OR Sweet'N*[tiab] OR "Swingle fruit"[tiab] OR thaumatin*[tiab] OR trichlorosucrose[tiab] OR trichlorosucrose [Supplementary Concept] OR trichlorogalactosucrose[tiab] OR trichlorogalacto-sucrose[tiab] OR Tri-Sweet[tiab] OR Trisweet[tiab] OR truvia[tiab] OR "sugar replacer*"[tiab] OR "sugar substitut*"[tiab] OR "sugar free"[tiab] OR "sugar-free"[tiab] OR "sugarfree"[tiab] OR "low sugar*"[tiab] OR "low-sugar*"[tiab] OR "zero sugar*" [tiab] OR "zero-sugar*"[tiab] OR "non-sugar*"[tiab] OR "non sugar*"[tiab] OR "no sugar*"[tiab] OR "no-sugar*"[tiab] OR "reduced sugar*"[tiab] OR "reduced-sugar*"[tiab] OR "artificially sweetened beverages"[mesh] OR "diet drink*"[tiab] OR "diet beverage*"[tiab] OR "diet soda*"[tiab] OR "diet soft drink*"[tiab] OR "diet cola*"[tiab]) AND (anthropometry[mesh] OR anthropometr*[tiab] OR "body weight"[mesh] OR "body weight*"[tiab] "bodyweight*"[tiab] OR "body size"[mesh] OR "body size*"[tiab] OR "body mass*"[tiab] OR "body mass index"[mesh] OR BMI[tiab] OR "Quetelet Index"[tiab] OR "Quetelet's Index"[tiab] OR "Quetelets Index"[tiab] OR "body composition"[mesh] OR "body composition*"[tiab] OR "Body Weights and Measures"[mesh] OR "body measure*"[tiab] OR "body weight maintenance"[mesh] OR "weight loss*"[tiab] OR "weight change*"[tiab] OR "weight gain*"[tiab] OR "weight maintenance*"[tiab] OR "weight control"[tiab] OR "weight increas*"[tiab] OR "weight reduc*"[tiab] OR slim*[tiab] OR adiposity[mesh] OR "adipose tissue"[mesh] OR adipos*[tiab] OR "fat tissue*"[tiab] OR "fatty tissue*"[tiab] OR "body fat*"[tiab] OR bodyfat[tiab] OR "body fat distribution"[mesh] OR "fat distribution*"[tiab] OR "fat pattern*"[tiab] OR "fat mass*"[tiab] OR "abdominal fat"[mesh] OR "abdominal fat*"[tiab] OR "subcutaneous fat"[mesh] OR "subcutaneous fat*"[tiab] OR "waist circumference*"[tiab] OR "waist circumference"[mesh] OR "Sagittal Abdominal Diameter"[mesh] OR "Sagittal Abdominal*"[tiab] OR "abdominal diameter*"[tiab] OR "abdominal height*"[tiab] OR "hip circumference*"[tiab] OR (("Waist-Hip"[tiab] OR "Waist to Hip"[tiab] OR "Waist-to-Hip"[tiab] OR "Waist-Height"[tiab] OR "Height-Waist"[tiab] OR "Waist to Height"[tiab] OR "Waist-to-Height"[tiab]) AND (Ratio*[tiab])) OR overweight[mesh] OR overweight[tiab] OR "over weight"[tiab] OR obesity[mesh] OR obes*[tiab] OR "corpulence"[tiab]) AND ("systematic review"[publication type] OR "systematic review"[tiab] OR "systematic reviews"[tiab] OR "systematic overview"[tiab] OR "meta analysis"[Publication Type] OR "meta analysis"[tiab] OR "meta-analysis"[tiab] OR "meta analyses"[tiab] OR "metaanalysis"[tiab] OR "metaanalyses"[tiab] OR "rapid review"[tiab] OR "scoping review" [tiab] OR "umbrella review"[tiab] OR "mapping review"[tiab] OR "overview of reviews"[tiab]) | English | 493 |
| **Scopus** | TITLE-ABS-KEY(*sweet* OR acesulf* OR acetosulfam* OR ace-K OR "ace K" OR advantame OR alitame OR aspartam* OR aspartylphenylalanine OR brazzein* OR canderel OR cyclamate* OR "cyclamic acid" OR Enliten OR "Equal" OR Goldswite OR grosvenorii OR hermesetas OR "Luo Han Guo" OR milisucre OR mogroside* OR monellin OR "monk fruit" OR neohesperidin OR neotame OR Nectresse OR Nozucar OR Newtame OR nutrasweet OR phyllodulcin OR PureLo OR "Pure Lo" OR PureVia OR "Pure Via" OR rebaudioside* OR rebuadiana OR rebiana OR rebaudianum OR rebaudianums OR saccharin* OR "Siraitia grosvenorii" OR splenda OR stevia OR stevias OR steviol* OR stevioside OR sucralose OR "Sugar Twin" OR Sunett OR Sweet'N* OR "swingle fruit" OR thaumatin OR trichlorosucrose OR trichlorogalactosucrose OR trichlorogalacto-sucrose OR Tri-Sweet OR Trisweet OR truvia OR "sugar replacer*" OR "sugar substitut*" OR "sugar free" OR "sugar-free" OR "sugarfree" OR "low sugar*" OR "low-sugar*" OR "zero sugar*" OR "zero-sugar*" OR "non-sugar*" OR "non sugar*" OR "no sugar*" OR "no-sugar*" OR "reduced sugar*" OR "reduced-sugar*" OR "diet drink*" OR "diet beverage*" OR "diet soda*" OR "diet soft drink*" OR "diet cola*") AND ( TITLE-ABS-KEY (anthropometr* OR "body weight*" OR "bodyweight*" OR "body size*" OR "body mass*" OR BMI OR "Quetelet Index" OR "Quetelet's Index" OR "Quetelets Index" OR "body composition*" OR "body measure*" OR "weight loss*" OR "weight change*" OR "weight gain*" OR "weight maintenance*" OR "weight control" OR "weight increas*" OR "weight reduc*" OR slim* OR adipos* OR "fat tissue*" OR "fatty tissue*" OR "body fat*" OR bodyfat OR "fat distribution*" OR "fat pattern*" OR "fat mass*" OR "abdominal fat*" OR "subcutaneous fat*" OR "waist circumference*" OR "Sagittal Abdominal*" OR "abdominal diameter*" OR "abdominal height*" OR "hip circumference*" OR ( ( "Waist-Hip" OR "Waist to Hip" OR "Waist-to-Hip" OR "Waist-Height" OR "Height-Waist" OR "Waist to Height" OR "Waist-to-Height" ) AND ( ratio*) ) OR overweight OR "over weight" OR obes* OR corpulence) ) AND TITLE-ABS-KEY ( "systematic review" OR "systematic reviews" OR "systematic overview" OR "meta analysis" OR "meta-analysis" OR "meta analyses" OR "metaanalysis" OR "metaanalyses" OR "rapid review" OR "scoping review" OR "umbrella review" OR "mapping review" OR "overview of reviews" ) | English | 782 |
| **Cochrane Library** | (*sweet* OR acesulf* OR acetosulfam* OR ace-K OR "ace K" OR advantame OR alitame OR aspartam* OR aspartylphenylalanine OR brazzein* OR canderel OR cyclamate* OR "cyclamic acid" OR Enliten OR "Equal" OR Goldswite OR grosvenorii OR hermesetas OR "Luo Han Guo" OR milisucre OR mogroside* OR monellin OR "monk fruit" OR neohesperidin OR neotame OR Nectresse OR Nozucar OR Newtame OR nutrasweet OR phyllodulcin OR PureLo OR "Pure Lo" OR PureVia OR "Pure Via" OR rebaudioside* OR rebuadiana OR rebiana OR rebaudianum OR rebaudianums OR saccharin* OR "Siraitia grosvenorii" OR splenda OR stevia OR stevias OR steviol* OR stevioside OR sucralose OR "Sugar Twin" OR Sunett OR Sweet'N* OR "swingle fruit" OR thaumatin OR trichlorosucrose OR trichlorogalactosucrose OR trichlorogalacto-sucrose OR Tri-Sweet OR Trisweet OR truvia OR "sugar replacer*" OR "sugar substitut*" OR "sugar free" OR "sugar-free" OR "sugarfree" OR "low sugar*" OR "low-sugar*" OR "zero sugar*" OR "zero-sugar*" OR "non-sugar*" OR "non sugar*" OR "no sugar*" OR "no-sugar*" OR "reduced sugar*" OR "reduced-sugar*" OR "diet drink*" OR "diet beverage*" OR "diet soda*" OR "diet soft drink*" OR "diet cola*") :ti,ab,kw AND (anthropometr* OR "body weight*" OR "bodyweight*" OR "body size*" OR "body mass*" OR BMI OR "Quetelet Index" OR "Quetelet's Index" OR "Quetelets Index" OR "body composition*" OR "body measure*" OR "weight loss*" OR "weight change*" OR "weight gain*" OR "weight maintenance*" OR "weight control" OR "weight increas*" OR "weight reduc*" OR slim* OR adipos* OR "fat tissue*" OR "fatty tissue*" OR "body fat*" OR bodyfat OR "fat distribution*" OR "fat pattern*" OR "fat mass*" OR "abdominal fat*" OR "subcutaneous fat*" OR "waist circumference*" OR "Sagittal Abdominal*" OR "abdominal diameter*" OR "abdominal height*" OR "hip circumference*" OR ( ( "Waist-Hip" OR "Waist to Hip" OR "Waist-to-Hip" OR "Waist-Height" OR "Height-Waist" OR "Waist to Height" OR "Waist-to-Height" ) AND ( ratio*) ) OR overweight OR "over weight" OR obes* OR corpulence ):ti,ab,kw | English; Cochrane Reviews or Cochrane Protocols | 61 |

^1^ Results from literature search conducted in November 2022.

## Supplemental searches in gray literature

Additional searches were conducted in November – December 2022 to identify publications in grey literature. These included searches of publications by the WHO (<https://www.who.int/publications/i>), FDA (<https://www.fda.gov/>), European Union (EU) Register of Health Claims (<https://ec.europa.eu/food/food-feed-portal/screen/health-claims/eu-register>), Great Britain Nutrition and Health Claims Register (<https://www.gov.uk/government/publications/great-britain-nutrition-and-health-claims-nhc-register>), Food Standards Australia New Zealand Nutrition, Health and Related Claims (<https://www.foodstandards.gov.au/industry/labelling/Pages/Nutrition-health-and-related-claims.aspx>), and Health Canada (<https://www.canada.ca/en/services/health/food-nutrition.html>).

## PI(E)COS Criteria

**Participants/population**

- **Included participants/populations:**
  - SR including generally healthy populations or with disease prevalent among Western populations (e.g., obesity, hypertension, hyperlipidemia, type 2 diabetes mellitus, metabolic syndrome).
  - No restriction on age.
- **Excluded participants/populations:**
  - SR exclusively among pregnant or lactating women.
  - SR exclusively among populations with chronic diseases (e.g., cancer, chronic kidney disease, chronic lung disease, heart disease, HIV/AIDS), metabolic disorders (e.g., irritable bowel syndrome, phenylketonuria, maple syrup urine disease), endocrine diseases (e.g., acromegaly, adrenal insufficiency, Addison’s disease, Cushing’s syndrome, cystic fibrosis, Graves’ disease, Hashimoto’s disease, hyperthyroidism, hypothyroidism, multiple endocrine neoplasia type 1, polycystic ovary syndrome, primary hyperparathyroidism, prolactinoma, Turner syndrome), or clinical nutrient deficiencies.
  - SR exclusively among populations with eating disorders (e.g., anorexia nervosa, bulimia nervosa, binge eating disorder), sensory disorders (e.g., ageusia, dysgeusia), or genetic disorders affecting food intake (e.g., Prader-Willi syndrome).
  - SR exclusively among bariatric surgery patients.
  - SR exclusively among populations taking medications known to affect sensory perception, appetite, or body weight (e.g., Acetazolamide, Amiodarone, Benzphetamine, Captopril, Cisplatin, Diethylpropion, Ephedrine, Eszopiclone, Liraglutide, Lithium, Lorcaserin, Maribavir, [Naltrexone-Bupropion](https://medlineplus.gov/druginfo/meds/a695033.html), Orlistat, Phendimetrazine, Phentermine, Phenylpropanolamine, Procainamide, Terbenafine, Topiramate).
  - SR exclusively among populations seeking treatment for drug or alcohol use disorders.

**Intervention(s), exposure(s)**

- **Included intervention(s), exposure(s):**
  - Intake of any type of LCS used alone or in combination with other foods, beverages, LCS, or nutritive sweeteners.
  - No restriction on intervention/exposure duration.
- **Excluded intervention(s), exposure(s):**
  - SR that investigated intake of sugar alcohols exclusively.
  - SR that investigated rare sugars exclusively.

**Comparator(s)/control**

- **Included comparator(s)/control:**
  - All comparators/controls included in SR, including lower dose or no LCS (e.g., sugar, placebo, no intervention/exposure) or comparison to other LCS.
- **Excluded comparator(s)/control:**
  - No comparator(s)/control were excluded.

**Outcomes**

- **Included outcomes**
  - Measures of adiposity, including body weight (kg), body mass index (BMI, kg/m^2^), prevalence of overweight/obesity, fat and lean body mass (kg), body fat percentage (%), waist circumference (cm), and/or waist: hip ratio effect estimates.
- **Excluded outcomes**
  - Energy intake
  - Any other outcomes not related to adiposity or body weight status.

**Study Design**

- **Types of studies included:**
  - SR on randomized controlled trials (RCT) and/or non-randomized studies (NRS).
  - Systematic searches of the literature (e.g., evidence maps, scoping reviews) that do not provide information on the results observed in the identified studies were identified but not further evaluated.
  - Systematic search that provides a narrative summary only with limited information on inclusion/exclusion criteria were identified but not further evaluated.
  - SR in which LCS is the comparator (e.g., a SR on SSB intervention/exposure compared to any comparator) were identified, but not evaluated further.
  - Overviews of reviews were identified. A search of the reference list was conducted to ensure that all relevant SR identified in the overview of reviews were included in our results. Overviews of reviews were not further evaluated.
- **Types of studies excluded:**
  - Narrative reviews, individual studies, SR on non-clinical data.
  - U.S. Food and Drug Administration (FDA) Generally Recognized as Safe (GRAS) notifications; FDA Food Additive Final Rules

## Data Extraction Template

The following table describes the data extracted from each eligible systematic review. All data was extracted independently by two reviewers with differences reconciled by a third reviewer.

| **Data Extracted** | **Description** |
| --- | --- |
| ***Overview*** |  |
| Primary aim of review | Text |
| Secondary aim of review (if applicable) | Text |
| What is the primary intervention/exposure investigated? | Any LCS, independent of vehicle |
| *Select one* | Specific LCS, independent of vehicle |
|  | LCS beverages only |
|  | Other intervention/exposure; LCS is the control |
|  | Other |
| Study design | Systematic Review only |
| *Select one* | Systematic Review and Meta-Analysis |
|  | Rapid Review |
|  | Scoping Review and/or Evidence Map |
|  | Overview of Reviews |
|  | Systematic search with narrative summary only |
|  | Protocol only |
|  | Erratum only |
|  | Other |
| Describe funding source(s) | Text |
| ***Inclusion/Exclusion Criteria*** |  |
| Population Inclusion Criteria | Text |
| Population Exclusion Criteria | Text |
| Intervention/Exposure Inclusion Criteria | Text |
| Intervention/Exposure Exclusion Criteria | Text |
| Comparator Inclusion Criteria | Text |
| Comparator Exclusion Criteria | Text |
| Outcomes Inclusion Criteria | Text |
| Outcomes Exclusion Criteria | Text |
| Study Design Inclusion Criteria | Text |
| Study Design Exclusion Criteria | Text |
| Setting Inclusion Criteria | Text |
| Setting Exclusion Criteria | Text |
| Duration Inclusion Criteria | Text |
| Duration Exclusion Criteria | Text |
| Other Inclusion Criteria | Text |
| Other Exclusion Criteria | Text |
| Sex Inclusion Criteria | Males only |
| *Select one* | Females only |
|  | Males and Females |
|  | Sex not an inclusion criterion |
| Health Status Inclusion Criteria | Generally healthy |
| *Select multiple* | Type 2 Diabetes |
|  | Hypertension |
|  | Hyperlipidemia |
|  | Metabolic Syndrome |
|  | Health status not an exclusion criterion |
|  | Not specified |
|  | Other |
| Body Weight Status Inclusion Criteria | Underweight |
| *Select multiple* | Normal weight |
|  | Overweight |
|  | Obese |
|  | Extremely obese |
|  | Body weight status not exclusion criteria |
|  | Not specified |
|  | Other |
| Country Study Conducted Inclusion Criteria | United States |
| *Select multiple* | UK |
|  | Canada |
|  | China |
|  | Australia |
|  | Country study conducted not an exclusion criterion |
|  | Not Specified |
|  | Other |
| Minimum Age (y) | Numeric |
| Maximum Age (y) | Numeric |
| Minimum Body weight (kg) | Numeric |
| Maximum Body weight (kg) | Numeric |
| Minimum BMI (kg/m^2^) | Numeric |
| Maximum BMI (kg/m^2^) | Numeric |
| Included LCS | Any LCS |
| *Select multiple* | Aspartame |
|  | Acesulfame-K |
|  | Saccharin |
|  | Sucralose |
|  | Stevia/Steviol Glycosides/Rebaudiosides |
|  | Sugar alcohols |
|  | Rare sugars |
|  | Unspecified mixture of LCS |
|  | Not specified |
|  | Other |
| Vehicle | LCS beverages |
| *Select multiple* | LCS foods |
|  | LCS packets |
|  | Capsules |
|  | Not specified |
|  | Other |
| Comparator | Any comparator |
| *Select multiple* | Sugar-sweetened beverages |
|  | Water |
|  | Sugar |
|  | No intervention/exposure |
|  | Lower dose of LCS |
|  | Other LCS |
|  | Other |
| Included Relevant Outcomes | BW |
| *Select multiple* | BMI |
|  | WC |
|  | W:H ratio |
|  | Body fat mass |
|  | Body fat percentage |
|  | Lean mass |
|  | Incident overweight/obesity |
|  | Incident obesity |
|  | Incident elevated WC |
|  | Other |
| List all other outcomes not relevant to this review. | Text |
| Included Study Designs | RCT, parallel arm |
| *Select multiple* | RCT, cross-over |
|  | RCT, not further specified |
|  | Non-randomized clinical trial |
|  | Longitudinal cohort |
|  | Case-control studies |
|  | Cross-sectional studies |
|  | Other |
| ***Literature Search Methods*** |  |
| Databases searched | MEDLINE/PubMed |
| *Select multiple* | Web of Science |
|  | Scopus |
|  | Cochrane Library/CENTRAL |
|  | EMBASE |
|  | CINAHL |
|  | Google Scholar |
|  | Other |
| Were there language restrictions on the literature search? | Yes |
| *Select one* | No |
|  | Not specified |
| Oldest Year Searched | Numeric.  Oldest restriction data on literature search. If no year cut off, type NA. If not specified, type NS. |
| Most Recent Year Searched | Numeric.  Most recent restriction data on literature search. If not specified, type NS. |
| Date of last search update | MM/DD/YYY.  List the date of last search. If they provide no information on whether an update was conducted, type NS. If they explicitly state they did not conduct an updated, type "no update". |
| Did the review search for unpublished data/gray literature? | Yes |
| *Select one* | No |
|  | Not specified |
|  | Other |
| Did the review report a replicable search strategy? | Yes, the search strategy is replicable |
| *Select one* | No, but key terms are reported |
|  | No |
|  | Other |
| What components were included in the search string? | Search string not reported |
| *Select multiple* | LCS terms, general |
|  | LCS terms specific |
|  | Other food/diet related terms |
|  | BW-related terms |
|  | Other health-related terms |
|  | Study design terms |
|  | Age terms |
|  | Other |
| ***Screening and Data Extraction Methods*** |  |
| Measures of effect extracted | Change from baseline |
| *Select multiple* | End |
|  | Not specified |
|  | Other |
| Method for screening of studies for eligibility | Completed in duplicate or more |
| *Select one* | Completed by 1 reviewer |
|  | Completed by 1 reviewer and a subset verified by a second reviewer |
|  | Not reported |
|  | Other |
| Method for data extraction from primary studies | Completed in duplicate or more |
| *Select one* | Completed by 1 reviewer |
|  | Completed by 1 reviewer and verified by a second reviewer |
|  | Completed by 1 reviewer with uncertainties verified by a second reviewer |
|  | Not reported |
|  | Other |
| How was missing data managed? | Study authors contacted |
| *Select multiple* | Study authors not contacted |
|  | Data imputed |
|  | Studies excluded |
|  | Not specified |
|  | Other |
| If data imputed, specify methods | Text |
| What covariates were included in the adjusted models from the studies included in the meta-analysis(es)? | Text |
| ***Analysis Methods*** |  |
| Method for the synthesis of results | Meta-analysis |
| *Select multiple* | Narrative summary |
|  | Tabular/graphical summary of results |
|  | Other |
| Among reviews without meta-analysis, was the decision to not perform meta-analysis explained in the review article? | Yes |
| *Select one* | No |
|  | Not applicable; meta-analysis conducted |
| If applicable, describe the decision to not perform a meta-analysis. | Text |
| If meta-analysis was conducted, what methods were used? | Fixed Effects |
| *Select multiple* | Random Effects |
|  | Dose-response |
|  | Random effects with significant or substantial heterogeneity, fixed effect otherwise |
|  | Meta-analysis not conducted |
|  | Other |
| Among reviews that did not conduct dose-response meta-analysis, was the decision to not conduct dose-response meta-analysis justified, either by the authors in the report or based on the question being investigated? | Dose-response meta-analysis conducted |
| *Select one* | Decision was justified |
|  | Decision was NOT justified |
|  | No meta-analysis conducted |
|  | Other |
| Describe strategies implemented to address heterogeneity. | Text |
| Were subgroup analyses conducted? | Yes |
| *Select one* | No |
|  | Not specified |
|  | Not applicable, meta-analysis not conducted |
|  | Other |
| If subgroup analyses were conducted, describe methods. | Text |
| Were multiple study design types pooled in the meta-analysis? | Yes |
| *Select one* | No |
|  | Not specified |
|  | Not applicable, meta-analysis not conducted |
|  | Other |
| If multiple study design types were pooled, describe methods. | Text |
| What methods were used to test for small study effects? | Egger's test |
| *Select multiple* | Visual inspection of funnel plot |
|  | Begg's test |
|  | No test for small study effects |
|  | Not applicable, meta-analysis not conducted |
|  | Other |
| ***Risk of Bias (ROB)/Study Quality Methods*** |  |
| Was ROB considered in analyses? | Yes |
| *Select one* | No |
|  | Not applicable, meta-analysis not conducted |
|  | Other |
| If ROB was considered, describe methods. | Text |
| Did the review cite a protocol? | Yes |
| *Select one* | No |
|  | Other |
| Did the review cite a reporting guideline? | PRISMA |
| *Select one* | MOOSE |
|  | No reporting guideline |
|  | Other |
| Method used to evaluate ROB | Cochrane ROB Tool |
| *Select multiple* | Cochrane ROB 2 Tool |
|  | JBI Critical Appraisal Checklist for Randomized Controlled Trials |
|  | Newcastle-Ottawa Scale |
|  | Risk of Bias in Non-randomized Studies of Interventions (ROBINS-I) |
|  | JBI Critical Appraisal Checklist for Quasi-Experimental Studies |
|  | AHRQ for Cross-Sectional Studies |
|  | Quality in Prognosis Studies |
|  | STROBE |
|  | AMSTAR |
|  | AMSTAR-2 |
|  | ROBIS |
|  | ROB not evaluated |
|  | Other |
| Method for the assessment of ROB among reviews that assessed ROB. | Completed in duplicate or more |
|  | Completed by 1 reviewer and verified by a second reviewer |
|  | Not reported |
|  | Risk of Bias was not assessed |
| What method was used to evaluate certainty of evidence? | GRADE |
| *Select one* | NutriGRADE |
|  | SIGN |
|  | NHMRC FORM Methodology |
|  | Certainty of evidence not evaluated |
|  | Other |
| Method for the assessment of quality of evidence among reviews that assessed quality of evidence. | Completed in duplicate or more |
| *Select one* | Completed by 1 reviewer and verified by a second reviewer |
|  | Not reported |
|  | Quality of evidence was not assessed. |
|  | Other |
| Was study compliance considered in the RoB/study quality evaluation? | Yes |
| *Select one* | No |
|  | Not applicable; NRS only |
|  | RoB not evaluated |
|  | Other |
| ***Results*** |  |
| Comparison X Figure/Table Number | Extracted for each meta-analysis conducted from 1 - X. |
| Comparison X Study Design | Extracted for each meta-analysis conducted from 1 - X. |
| Comparison X Population | Extracted for each meta-analysis conducted from 1 - X. |
| Comparison X Intervention/Exposure | Extracted for each meta-analysis conducted from 1 - X. |
| Comparison X Comparator | Extracted for each meta-analysis conducted from 1 - X. |
| Comparison X Outcome | Extracted for each meta-analysis conducted from 1 - X. |
| Comparison X Number of studies | Extracted for each meta-analysis conducted from 1 - X. |
| Comparison X Number of participants | Extracted for each meta-analysis conducted from 1 - X. |
| Comparison X MD | Extracted for each meta-analysis conducted from 1 - X. |
| Comparison X MD Lower 95% CI | Extracted for each meta-analysis conducted from 1 - X. |
| Comparison X MD Upper 95% CI | Extracted for each meta-analysis conducted from 1 - X. |
| Comparison X SMD | Extracted for each meta-analysis conducted from 1 - X. |
| Comparison X SMD Lower 95% CI | Extracted for each meta-analysis conducted from 1 - X. |
| Comparison X SMD Upper 95% CI | Extracted for each meta-analysis conducted from 1 - X. |
| Comparison X I2 (%) | Extracted for each meta-analysis conducted from 1 - X. |
| Comparison X Reported effect p-value | Extracted for each meta-analysis conducted from 1 - X. |
| Comparison X Stat sig? | Extracted for each meta-analysis conducted from 1 - X. |
| Comparison X Cut off for stat sig | Extracted for each meta-analysis conducted from 1 - X. |
| Comparison X Favors LCS or control | Extracted for each meta-analysis conducted from 1 - X. |
| Comparison X Summary Result Text | Extracted for each meta-analysis conducted from 1 - X. |
| Among meta-analyses that tested for small study effects, was there evidence of small study effects? | Yes |
| *Select one* | No |
|  | Not reported |
|  | Tests for small study effects not conducted |
|  | Not applicable, meta-analysis not conducted |
| Among meta-analyses with evidence of small study effects, were results adjusted for small study effects? | No evidence of small study effects |
| *Select one* | No |
|  | Yes, using trim and fill |
|  | Yes, a study was excluded |
|  | Tests for small study effects not conducted |
|  | Not applicable, meta-analysis not conducted |
| Text discussion of small study effects. | Text |
| Describe sources of heterogeneity identified by review authors. | Text |
| Text discussing potential sources of bias of included studies. | Text |
| Text discussing potential methodological quality concerns of included studies. | Text |
| Did the review consider ROB of primary studies in the interpretation of results? | Yes, risk of bias is acknowledged as a limitation |
| *Select one* | Yes, bias is described as unlikely to have affected findings |
|  | No, risk of bias is not discussed |
|  | Other |
| If ROB was considered, describe. | Text |
| Did the review consider consistency in the interpretation of results? | Yes, consistency across primary studies is used to support findings |
| *Select one* | Yes, inconsistency across primary studies is acknowledged or described as a limitation |
|  | No, consistency is not discussed |
|  | Other |
| If consistency was considered, describe. | Text |
| Did the review consider directness in the interpretation of results? | Yes, directness across primary studies is used to support findings |
| *Select one* | Yes, indirectness across primary studies is acknowledged or described as a limitation |
|  | No, indirectness is not discussed |
|  | Other |
| If directness was considered, describe. | Text |
| Did the review consider precision in the interpretation of results? | Yes, precise results, large sample size, or a large number of events is used to support findings |
| *Select one* | Yes, imprecision across primary studies is acknowledged or described as a limitation |
|  | No, precision is not discussed |
|  | Other |
| If precision was considered, describe. | Text |
| Did the review consider the potential for publication bias in the interpretation of results? | Yes, the potential for publication bias is acknowledged as a limitation |
| *Select one* | Yes, publication bias is described as unlikely to have affected findings |
|  | No, publication bias is not discussed |
|  | Other |
| If publication bias was considered, describe. | Text |
| Other potential sources of bias noted by review authors. | Text |
| ***Conclusions*** |  |
| Summary X Population | Extracted for each certainty of evidence conducted from 1 - X. |
| Summary X Settings | Extracted for each certainty of evidence conducted from 1 - X. |
| Summary X Study Design | Extracted for each certainty of evidence conducted from 1 - X. |
| Summary X Intervention | Extracted for each certainty of evidence conducted from 1 - X. |
| Summary X Comparison | Extracted for each certainty of evidence conducted from 1 - X. |
| Summary X Outcome | Extracted for each certainty of evidence conducted from 1 - X. |
| Summary X Estimated Effect | Extracted for each certainty of evidence conducted from 1 - X. |
| Summary X Certainty of Evidence (GRADE) | Extracted for each certainty of evidence conducted from 1 - X. |
| Summary X ROB | Extracted for each certainty of evidence conducted from 1 - X. |
| Summary X Consistency | Extracted for each certainty of evidence conducted from 1 - X. |
| Summary X Directness | Extracted for each certainty of evidence conducted from 1 - X. |
| Summary X Precision | Extracted for each certainty of evidence conducted from 1 - X. |
| Summary X Evidence of Publication Bias | Extracted for each certainty of evidence conducted from 1 - X. |
| Summary X Comments | Extracted for each certainty of evidence conducted from 1 - X. |
| What limitations were described by the study authors? | Text |
| Additional limitations/concerns NOT described by the study authors? | Text |
| What future directions were proposed by the study authors? | Text |
| Conclusion statement | Text |
| What was the authors' conclusion regarding the association between LCS and BW? | Decrease BW/more beneficial |
| *Select one* | Neutral (no directional effect or association) |
|  | Increase BW/less beneficial |
|  | Evidence is insufficient to draw a conclusion |
|  | No conclusion directly relevant to the LCS–BW relationship |
|  | Unable to draw a conclusion from the paper |
|  | Other |

# **Supplemental Tables**

## Supplemental Table 1. List of excluded studies based on full text review

| **Reference** | **Reason for exclusion** |
| --- | --- |
| Ángeles Pérez-Ara M, Gili M, Visser M, Penninx BWJH, Brouwer IA, Watkins E, Owens M, García-Toro M, Hegerl U, Kohls E, Bot M, Roca M. Associations of non-alcoholic beverages with major depressive disorder history and depressive symptoms clusters in a sample of overweight adults. Nutrients 2020;12(10):3202. | Excluded study design |
| ACNP 59th Annual Meeting: Poster Session II. Neuropsychopharmacology 2020;45:170-277. | Multiple studies; no one study met all inclusion criteria |
| Agras WS, Mascola AJ. Risk factors for childhood overweight. Current Opinion in Pediatrics 2005;17(5):648-652. | Excluded study design |
| Anderson G H, Foreyt J, Sigman-Grant M, Allison D B. The use of low-calorie sweeteners by adults: impact on weight management. J Nutr 2012;142(6):1163s-9s. | Excluded study design |
| Androutsos O, Charmandari E. Determinants, Screening, Prevention and Management of Obesity in Youth: New Evidence and Horizons. Nutrients 2022;14(16):3280. | Excluded study design |
| Archibald Alyssa J, Dolinsky Vernon W, Azad Meghan B. Early-Life Exposure to Non-Nutritive Sweeteners and the Developmental Origins of Childhood Obesity: Global Evidence from Human and Rodent Studies. Nutrients 2018;10(2):194. | Excluded study design |
| Aune D. Soft drinks, aspartame, and the risk of cancer and cardiovascular disease. American Journal of Clinical Nutrition 2012;96(6):1249-1251. | Excluded study design |
| Baker-Smith Carissa M, de Ferranti Sarah D, Cochran William J, Committee on Nutrition, Section on Gastroenterology, Hepatology, Nutrition, Abrams Steven A, Fuchs George J III, Kim Jae Hong, Lindsey C Wesley, Magge Sheela N, Rome Ellen S, Schwarzenberg Sarah Jane, Lightdale Jenifer R, Brumbaugh David, Cohen Mitchell B, Dotson Jennifer L, Harpavat Sanjiv, Oliva-Hemker Maria M, Heitlinger Leo A. The Use of Nonnutritive Sweeteners in Children. Pediatrics 2019;144(5):e20192765. | Excluded study design |
| Baker-Smith CM, De Ferranti SD, Cochran WJ. The use of nonnutritive sweeteners in children. Pediatrics 2019;144(5):e20192765. | Excluded study design |
| Battault S, Pallot F, Risdon S, Meyer G, Walther G. Non-nutritive sweeteners: Mechanisms of action, effects on eating behavior and glycemic control. Medecine des Maladies Metaboliques 2022;16(6):527-536. | Publication not in English |
| Bellisle F, Drewnowski A. Intense sweeteners, energy intake and the control of body weight. Eur J Clin Nutr 2007;61(6):691-700. | Excluded study design |
| Bellisle F, Perez C. Low-energy substitutes for sugars and fats in the human diet: impact on nutritional regulation. Neurosci Biobehav Rev 1994;18(2):197-205. | Excluded study design |
| Bellisle F. Intense Sweeteners, Appetite for the Sweet Taste, and Relationship to Weight Management. Curr Obes Rep 2015;4(1):106-10. | Excluded study design |
| Benton D. Can artificial sweeteners help control body weight and prevent obesity? Nutr Res Rev 2005;18(1):63-76. | Excluded outcome |
| Bes-Rastrollo M, Martinez-Gonzalez MA. Concerns about the Discretion of Sweetened Beverages. Journal of the American Dietetic Association 2009;109(3):404-5. | Excluded study design |
| Bes-Rastrollo M, Martinez-Gonzalez MA. Differential underreporting and other caveats about sugar-sweetened beverages and weight gain. American Journal of Clinical Nutrition 2008;88(5):1450-1451. | Excluded study design |
| Bhagavathula AS, Rahmani J, Vidyasagar K, Tesfaye W, Khubchandani J. Sweetened beverage consumption and risk of cardiovascular mortality: A systematic review and meta-analysis. Diabetes and Metabolic Syndrome: Clinical Research and Reviews 2022;16(4):102462. | Excluded outcome |
| Borges MC, Louzada ML, de Sá TH, Laverty AA, Parra DC, Garzillo JMF, Monteiro CA, Millett C. Artificially Sweetened Beverages and the Response to the Global Obesity Crisis. PLoS Medicine 2017;14(1):e1002195. | Excluded study design |
| Borys J-M, De Ruyter JC, Finch H, Harper P, Levy E, Mayer J, Richard P, Du Plessis HR, Seidell JC, Vinck J. Hydration and obesity prevention. Obesity Facts 2014;7(SUPPL. 2):37-48. | Excluded study design |
| Bray GA. Energy and fructose from beverages sweetened with sugar or high-fructose corn syrup pose a health risk for some people. Advances in Nutrition 2013;4(2):220-225. | Excluded study design |
| Bray GA. Epidemiologic and mechanistic studies of sucrose and fructose in beverages and their relation to obesity and cardiovascular risk. Nutrition and Cardiometabolic Health 2017237-249. | Excluded study design |
| Bray GA. Low-Carbohydrate Diets and Realities of Weight Loss. JAMA 2003;289(14):1853-1855. | Excluded study design |
| Brown R J, Rother K I. Non-nutritive sweeteners and their role in the gastrointestinal tract. J Clin Endocrinol Metab 2012;97(8):2597-605. | Excluded study design |
| Burke M V, Small D M. Physiological mechanisms by which non-nutritive sweeteners may impact body weight and metabolism. Physiol Behav 2015;152(Pt B):381-8. | Excluded study design |
| Cai C, Sivak A, Davenport M H. Effects of prenatal artificial sweeteners consumption on birth outcomes: a systematic review and meta-analysis. Public Health Nutr 2021;24(15):5024-5033. | Excluded population |
| Cavagnari B M. Non-caloric sweeteners and body weight. Medicina (B Aires) 2019;79(2):115-122. | Excluded study design |
| Chen L. Sugar-Sweetened Beverages and Cardiovascular Disease. Current Nutrition Reports 2012;1(2):109-114. | Excluded study design |
| Cheungpasitporn W, Thongprayoon C, Edmonds P J, Srivali N, Ungprasert P, Kittanamongkolchai W, Erickson S B. Sugar and artificially sweetened soda consumption linked to hypertension: a systematic review and meta-analysis. Clin Exp Hypertens 2015;37(7):587-93. | Excluded outcome |
| Costa CS, Del-Ponte B, Assunção MCF, Santos IS. Consumption of ultra-processed foods and body fat during childhood and adolescence: A systematic review. Public Health Nutrition 2018;21(1):148-159. | Excluded intervention/exposure |
| Cozma AI, Sievenpiper JL. The role of fructose, sucrose, and high-fructose corn syrup in diabetes. European Endocrinology 2013;10(1):51-60. | Excluded study design |
| Daher MI, Matta JM, Abdel Nour AM. Non-nutritive sweeteners and type 2 diabetes: Should we ring the bell? Diabetes Research and Clinical Practice 2019;155:107786. | Excluded outcome |
| Drewnowski A. Intense sweeteners and energy density of foods: implications for weight control. Eur J Clin Nutr 1999;53(10):757-63. | Excluded study design |
| Drewnowski A. Intense sweeteners and the control of appetite. Nutr Rev 1995;53(1):44933. | Excluded study design |
| Drouin-Chartier J-P, Zheng Y, Li Y, Malik V, Pan A, Bhupathiraju SN, Tobias DK, Manson JE, Willett WC, Hu FB. Changes in consumption of sugary beverages and artificially sweetened beverages and subsequent risk of type 2 diabetes: Results from three large prospective U.S. Cohorts of women and men. Diabetes Care 2019;42(12):2181-2189. | Excluded study design |
| Dyson PA, Twenefour D, Breen C, Duncan A, Elvin E, Goff L, Hill A, Kalsi P, Marsland N, McArdle P, Mellor D, Oliver L, Watson K. Diabetes UK evidence-based nutrition guidelines for the prevention and management of diabetes. Diabetic Medicine 2018;35(5):541-547. | Excluded outcome |
| Ebrahimpour-koujan S, Saneei P, Larijani B, Esmaillzadeh A. Consumption of sugar-sweetened beverages and serum uric acid concentrations: a systematic review and meta-analysis. Journal of Human Nutrition and Dietetics 2021;34(2):305-313. | Excluded outcome |
| Ebrahimzadeh Attari Vahideh, Ardalan Mohammad Reza, Malek Mahdavi Aida, Gorbani Abolfazl. A review of the health hazards of artificial sweeteners: are they safe? Progress in Nutrition 2018;20(2-S):36-43. | Excluded study design |
| EFSA Panel on Dietetic Products Nutrition, Allergies. Scientific Opinion on the substantiation of health claims related to intense sweeteners and contribution to the maintenance or achievement of a normal body weight (ID 1136, 1444, 4299), reduction of post‐prandial glycaemic responses (ID 4298), maintenance of normal blood glucose concentrations (ID 1221, 4298), and maintenance of tooth mineralisation by decreasing tooth demineralisation (ID 1134, 1167, 1283) pursuant to Article 13 (1) of Regulation (EC) No 1924/2006. EFSA Journal 2011;9(6):2229. | Excluded study design |
| EFSA Panel on Dietetic Products Nutrition, Allergies. Scientific Opinion on the substantiation of health claims related to sugar free chewing gum and dental and oral health, including gum and tooth protection and strength (ID 1149), plaque acid neutralisation (ID 1150), maintenance of tooth mineralisation (ID 1151), reduction of oral dryness (ID 1240), and maintenance of the normal body weight (ID 1152) pursuant to Article 13 (1) of Regulation (EC) No 1924/2006. EFSA Journal 2009;7(10):1271. | Excluded study design |
| EFSA Panel on Food Additives, Nutrient Sources added to Food. Scientific Opinion on the re-evaluation of aspartame (E 951) as a food additive. EFSA Journal 2013;11(12):3496. | Excluded outcome |
| EFSA Panel on Food Additives, Nutrient Sources added to Food. Scientific Opinion on the safety of advantame for the proposed uses as a food additive. EFSA Journal 2013;11(7):3301. | Excluded study design |
| EFSA Panel on Food Additives, Nutrient Sources added to Food. Scientific Opinion on the safety of steviol glycosides for the proposed uses as a food additive. EFSA Journal 2010;8(4):1537. | Excluded study design |
| EFSA Panel on Food Additives, Nutrient Sources added to Food. Scientific opinion on the safety of the extension of use of steviol glycosides (E 960) as a food additive. EFSA Journal 2015;13(6):4146. | Excluded study design |
| EFSA Panel on Food Additives, Nutrient Sources added to Food. Scientific Opinion on the safety of the extension of use of thaumatin (E 957). EFSA Journal 2015;13(11):4290. | Excluded study design |
| EFSA Panel on Food Additives, Nutrient Sources added to Food. Scientific opinion on the safety of the proposed amendment of the specifications for steviol glycosides (E 960) as a food additive. EFSA Journal 2015;13(12):4316. | Excluded study design |
| European Food Safety Authority. Neotame as a sweetener and flavour enhancer - Scientific Opinion of the Panel on Food Additives, Flavourings, Processing Aids and Materials in Contact with Food. EFSA Journal 2007;5(11):581. | Excluded study design |
| Fernstrom J D. Non-nutritive sweeteners and obesity. Annu Rev Food Sci Technol 2015;6:119-36. | Excluded study design |
| Ferreira A V, Generoso S V, Teixeira A L. Do low-calorie drinks 'cheat' the enteral-brain axis? Curr Opin Clin Nutr Metab Care 2014;17(5):465-70. | Excluded study design |
| Forshee RA, Anderson PA, Storey ML. Sugar-sweetened beverages and body mass index in children and adolescents: A meta-analysis (American Journal of Clinical Nutrition (2008) 87 (1662-1671)). American Journal of Clinical Nutrition 2009;89(1):441. | Erratum of relevant publication |
| Fowler S P G. Low-calorie sweetener use and energy balance: Results from experimental studies in animals, and large-scale prospective studies in humans. Physiol Behav 2016;164(Pt B):517-523. | Excluded study design |
| Freswick P N. Artificial Sweetened Beverages and Pediatric Obesity: The Controversy Continues. Children (Basel) 2014;1(1):31-9. | Excluded study design |
| Garcia-Oropesa EM, Martinez-Lopez YE, Ruiz-Cejudo SM, Martínez-Ezquerro JD, Diaz-Badillo A, Ramirez-Pfeiffer C, Bustamante-Fuentes A, Lopez-Sosa EB, Moctezuma-Chavez OO, Nava-Gonzalez EJ, Perales-Torres AL, Perez-Navarro LM, Rosas-Diaz M, Carter K, Tapia B, Lopez-Alvarenga JC. Looking for Crumbs in the Obesity Forest: Anti-obesity Interventions and Obesity-Associated Cardiometabolic Traits in the Mexican Population. History and Systematic Review With Meta-Analyses. Frontiers in Medicine 2021;8. | Excluded intervention/exposure |
| Gardner C. Non-nutritive sweeteners: evidence for benefit vs. risk. Curr Opin Lipidol 2014;25(1):80-4. | Excluded study design |
| Goubgou M, Songré-Ouattara LT, Bationo F, Lingani-Sawadogo H, Traoré Y, Savadogo A. Biscuits: a systematic review and meta-analysis of improving the nutritional quality and health benefits. Food Production, Processing and Nutrition 2021;3(1). | Excluded intervention/exposure |
| Gougeon Réjeanne, Spidel Mark, Lee Kristy, Field Catherine J. Canadian Diabetes Association National Nutrition Committee Technical Review: Non-nutritive Intense Sweeteners in Diabetes Management 2004. | Excluded study design |
| Green C H, Syn W K. Non-nutritive sweeteners and their association with the metabolic syndrome and non-alcoholic fatty liver disease: a review of the literature. Eur J Nutr 2019;58(5):1785-1800. | Excluded study design |
| Greenwood DC, Threapleton DE, Evans CEL, Cleghorn CL, Nykjaer C, Woodhead C, Burley VJ. Association between sugar-sweetened and artificially sweetened soft drinks and type 2 diabetes: Systematic review and dose-response meta-analysis of prospective studies. British Journal of Nutrition 2014;112(5):725-734. | Excluded outcome |
| Hill JO. What do you say when your patients ask whether low-calorie sweeteners help with weight management? American Journal of Clinical Nutrition 2014;100(3):739-740. | Excluded study design |
| Hunter S R, Reister E J, Cheon E, Mattes R D. Low Calorie Sweeteners Differ in Their Physiological Effects in Humans. Nutrients 2019;11(11):2717. | Excluded study design |
| Iizuka K. Is the Use of Artificial Sweeteners Beneficial for Patients with Diabetes Mellitus? The Advantages and Disadvantages of Artificial Sweeteners. Nutrients 2022;14(21):4446. | Excluded study design |
| Imamura F, O'Connor L, Ye Z, Mursu J, Hayashino Y, Bhupathiraju SN, Forouhi NG. Consumption of sugar sweetened beverages, artificially sweetened beverages, and fruit juice and incidence of type 2 diabetes: Systematic review, meta-analysis, and estimation of population attributable fraction. BMJ (Online) 2015;351:h3576. | Excluded outcome |
| Imamura F, O'Connor L, Ye Z, Mursu J, Hayashino Y, Bhupathiraju SN, Forouhi NG. Consumption of sugar sweetened beverages, artificially sweetened beverages, and fruit juice and incidence of type 2 diabetes: Systematic review, meta-analysis, and estimation of population attributable fraction. British Journal of Sports Medicine 2016;50(8):496-504. | Excluded outcome |
| International Journal of Obesity: Introduction. International Journal of Obesity 2009;33(SUPPL. 3):S1-S88. | Excluded study design |
| Johnson R K, Lichtenstein A H, Anderson C A M, Carson J A, Després J P, Hu F B, Kris-Etherton P M, Otten J J, Towfighi A, Wylie-Rosett J. Low-Calorie Sweetened Beverages and Cardiometabolic Health: A Science Advisory From the American Heart Association. Circulation 2018;138(9):e126-e140. | Excluded study design |
| Kakleas K, Christodouli F, Karavanaki K. Nonalcoholic fatty liver disease, insulin resistance, and sweeteners: a literature review. Expert Review of Endocrinology and Metabolism 2020;15(2):83-93. | Excluded outcome |
| Khan TA, Sievenpiper JL. Controversies about sugars: results from systematic reviews and meta-analyses on obesity, cardiometabolic disease and diabetes. European Journal of Nutrition 2016;55:25-43. | Excluded study design |
| Kim Y, Je Y. Prospective association of sugar-sweetened and artificially sweetened beverage intake with risk of hypertension. Archives of Cardiovascular Diseases 2016;109(4):242-253. | Excluded outcome |
| Köhler K, Eksin M, Peil E, Sammel A, Uuetoa M, Villa I. Reducing the consumption of sugar-sweetened beverages and their negative health impact in Estonia: EVIPNet evidence brief for policy, number 1. 2017;(WHO/EURO:2017-3012-42770-59676). | Excluded outcome |
| Koplin J J, Kerr J A, Lodge C, Garner C, Dharmage S C, Wake M, Allen K J. Infant and young child feeding interventions targeting overweight and obesity: A narrative review. Obes Rev 2019;20 Suppl 1:31-44. | Excluded intervention/exposure |
| Laviada-Molina H, Molina-Seguí F, Arjona-Villicana RD, Morales-Gual M, Cuello-García CA, Pérez-Gaxiola G. Non-nutritive sweeteners for the prevention or treatment of being overweight or obesity. Cochrane Database of Systematic Reviews 2016;2016(8). | Protocol of included publication |
| Lee D, Chiavaroli L, Ayoub-Charette S, Khan TA, Zurbau A, Au-Yeung F, Cheung A, Liu Q, Qi X, Ahmed A, Choo VL, Blanco Mejia S, Malik VS, El-Sohemy A, de Souza RJ, Wolever TMS, Leiter LA, Kendall CWC, Jenkins DJA, Sievenpiper JL. Important Food Sources of Fructose-Containing Sugars and Non-Alcoholic Fatty Liver Disease: A Systematic Review and Meta-Analysis of Controlled Trials. Nutrients 2022;14(14):2846. | Excluded outcome |
| Levitsky DA, Sewall A, Zhong Y, Barre L, Shoen S, Agaronnik N, LeClair J-L, Zhuo W, Pacanowski C. Quantifying the imprecision of energy intake of humans to compensate for imposed energetic errors: A challenge to the physiological control of human food intake. Appetite 2019;133:337-343. | Excluded outcome |
| Litvak J, Parekh N, Deierlein A. Prenatal dietary exposures and offspring body size from 6 months to 18 years: A systematic review. Paediatric and Perinatal Epidemiology 2020;34(2):171-189. | Excluded population |
| Lo W-C, Ou S-H, Chou C-L, Chen J-S, Wu M-Y, Wu M-S. Sugar- and artificially-sweetened beverages and the risks of chronic kidney disease: a systematic review and dose-response meta-analysis. Journal of Nephrology 2021;34(6):1791-1804. | Excluded outcome |
| Lohner S, Kuellenberg de Gaudry D, Toews I, Ferenci T, Meerpohl JJ. Effects of non-nutritive sweeteners on diabetes: Reply to Laviada-Molina et al.. Diabetic Medicine 2021;38(9):e14589. | Excluded study design |
| Lohner S, Toews I, Kuellenberg de Gaudry D, Sommer H, Meerpohl JJ. Non-nutritive sweeteners for diabetes mellitus. Cochrane Database of Systematic Reviews 2017;2017(11). | Protocol of included publication |
| MacLeod J, Franz MJ, Handu D, Gradwell E, Brown C, Evert A, Reppert A, Robinson M. Academy of Nutrition and Dietetics Nutrition Practice Guideline for Type 1 and Type 2 Diabetes in Adults: Nutrition Intervention Evidence Reviews and Recommendations. Journal of the Academy of Nutrition and Dietetics 2017;117(10):1637-1658. | Excluded study design |
| Malik VS, Hu FB. Sugar-sweetened beverages and health: Where does the evidence stand? American Journal of Clinical Nutrition 2011;94(5):1161-1162. | Excluded study design |
| Malik VS, Willett WC, Hu FB. Sugar-sweetened beverages and BMI in children and adolescents: Reanalyses of a meta-analysis. American Journal of Clinical Nutrition 2009;89(1):438-439. | Excluded study design |
| Manavalan D, Shubrook C, Young CF. Consumption of Non-nutritive Sweeteners and Risk for Type 2 Diabetes: What Do We Know, and Not? Current Diabetes Reports 2021;21(12):53. | Excluded study design |
| Mattes R D, Popkin B M. Nonnutritive sweetener consumption in humans: effects on appetite and food intake and their putative mechanisms. Am J Clin Nutr 2009;89(1):44940. | Excluded study design |
| Mekary RA. Breakfast Skipping and Type 2 Diabetes: Where Do We Stand? Journal of Nutrition 2019;149(1):44929. | Excluded study design |
| Miranda C. Influence of ultra-processed foods consumption during pregnancy on baby's anthropometric measurements, from birth to the first year of life: A systematic review. Revista Brasileira de Saude Materno Infantil 2021;21(1). | Excluded population |
| Moona MM, Smits R, Kertesz J, Meyer A, Mackler L. Do complementary agents lower HbA1c when used with standard type 2 diabetes therapy? Journal of Family Practice 2014;63(6):336-338. | Excluded study design |
| Mooradian AD, Smith M, Tokuda M. The role of artificial and natural sweeteners in reducing the consumption of table sugar: A narrative review. Clinical Nutrition ESPEN 2017;18:44934. | Excluded study design |
| Muli S, Goerdten J, Oluwagbemigun K, Floegel A, Schmid M, Nöthlings U. A systematic review of metabolomic biomarkers for the intake of sugar-sweetened and low-calorie sweetened beverages. Metabolites 2021;11(8):546. | Excluded outcome |
| Nadolsky KZ. COUNTERPOINT: Artificial Sweeteners for Obesity-Better than Sugary Alternatives; Potentially a Solution. Endocrine Practice 2021;27(10):1056-1061. | Excluded study design |
| Narain A, Kwok C S, Mamas M A. Soft drink intake and the risk of metabolic syndrome: AÂ systematic review and meta-analysis. Int J Clin Pract 2017;71(2):e12927. | Excluded outcome |
| Narain A, Kwok C S, Mamas M A. Soft drinks and sweetened beverages and the risk of cardiovascular disease and mortality: a systematic review and meta-analysis. Int J Clin Pract 2016;70(10):791-805. | Excluded outcome |
| Naumann J, Biehler D, Lüty T, Sadaghiani C. Prevention and therapy of type 2 diabetes- what is the potential of daily water intake and its mineral nutrients? Nutrients 2017;9(8):914. | Excluded outcome |
| Nichol AD, Holle MJ, An R. Glycemic impact of non-nutritive sweeteners: A systematic review and meta-Analysis of randomized controlled trials. European Journal of Clinical Nutrition 2018;72(6):796-804. | Excluded outcome |
| Nordmann Herve. Sweeteners-Low calorie foods, beverages and sweeteners. Can they really contribute to a healthier future? (Part 1). Agro Food Industry Hi Tech 2012;23(1):27. | Excluded study design |
| Nutrition recommendations and interventions for diabetes: A position statement of the American Diabetes Association. Diabetes Care 2007;30(SUPPL. 1):S48-S65. | Excluded outcome |
| Onakpoya I J, Heneghan C J. Effect of the natural sweetener, steviol glycoside, on cardiovascular risk factors: a systematic review and meta-analysis of randomised clinical trials. Eur J Prev Cardiol 2015;22(12):1575-87. | Excluded outcome |
| Osei-Assibey G, Dick S, MacDiarmid J, Semple S, Reilly JJ, Ellaway A, Cowie H, McNeill G. The influence of the food environment on overweight and obesity in young children: A systematic review. BMJ Open 2012;2(6):e001538. | Excluded intervention/exposure |
| Pagliai G, Giangrandi I, Dinu M, Sofi F, Colombini B. Nutritional Interventions in the Management of Fibromyalgia Syndrome. Nutrients 2020;12(9):2525. | Excluded study design |
| Pan A, Hu FB. Question about a recent meta-analysis of lowcalorie sweeteners and body weight. American Journal of Clinical Nutrition 2014;100(6):1604. | Excluded study design |
| Pan A, Malik VS, Hao T, Willett WC, Mozaffarian D, Hu FB. Changes in water and beverage intake and long-term weight changes: Results from three prospective cohort studies. International Journal of Obesity 2013;37(10):1378-1385. | Excluded study design |
| Pepino M Y, Bourne C. Non-nutritive sweeteners, energy balance, and glucose homeostasis. Curr Opin Clin Nutr Metab Care 2011;14(4):391-5. | Excluded study design |
| Pepino M Y. Metabolic effects of non-nutritive sweeteners. Physiol Behav 2015;152(Pt B):450-5. | Excluded study design |
| Pérez-Morales ME, Bacardí-Gascón M, Jiménez-Cruz A. Childhood overweight and obesity prevention interventions among Hispanic children in the United States; systematic review. Nutricion Hospitalaria 2012;27(5):1415-1421. | Excluded intervention/exposure |
| Peters JC, Beck J. Low Calorie Sweetener (LCS) use and energy balance. Physiology and Behavior 2016;164:524-528. | Excluded study design |
| Popkin BM, Armstrong LE, Bray GM, Caballero B, Frei B, Willett WC. A new proposed guidance system for beverage consumption in the United States. American Journal of Clinical Nutrition 2006;83(3):529-542. | Excluded study design |
| Popkin BM, Malik V, Hu FB. Beverage: Health Effects. Encyclopedia of Food and Health 2015372-380. | Excluded study design |
| Qi X, Chiavaroli L, Lee D, Ayoub-Charette S, Khan TA, Au-Yeung F, Ahmed A, Cheung A, Liu Q, Blanco Mejia S, Choo VL, de Souza RJ, Wolever TMS, Leiter LA, Kendall CWC, Jenkins DJA, Sievenpiper JL. Effect of Important Food Sources of Fructose-Containing Sugars on Inflammatory Biomarkers: A Systematic Review and Meta-Analysis of Controlled Feeding Trials. Nutrients 2022;14(19):3986. | Excluded outcome |
| Raben A, Richelsen B. Artificial sweeteners: a place in the field of functional foods? Focus on obesity and related metabolic disorders. Curr Opin Clin Nutr Metab Care 2012;15(6):597-604. | Excluded study design |
| Redondo-Useros N, Nova E, González-Zancada N, Díaz LE, Gómez-Martínez S, Marcos A. Microbiota and lifestyle: A special focus on diet. Nutrients 2020;12(6):1776. | Excluded outcome |
| Renwick AG, Nordmann H. First European conference on aspartame: Putting safety and benefits into perspective. Synopsis of presentations and conclusions. Food and Chemical Toxicology 2007;45(7):1308-1313. | Excluded study design |
| Renwick AG. Intense sweeteners, food intake, and the weight of a body of evidence. Physiology and Behavior 1994;55(1):139-143. | Excluded study design |
| Ribeiro J. Diastolic dysfunction and type 1 diabetes: A sweet link? Revista Portuguesa de Cardiologia 2021;40(10):767-769. | Excluded study design |
| Roberts J R. The paradox of artificial sweeteners in managing obesity. Curr Gastroenterol Rep 2015;17(1):423. | Excluded study design |
| Rogers PJ, Appleton KM. Correction: The effects of low-calorie sweeteners on energy intake and body weight: a systematic review and meta-analyses of sustained intervention studies (International Journal of Obesity, (2021), 45, 3, (464-478), 10.1038/s41366-020-00704-2). International Journal of Obesity 2021;45(9):2139-2140. | Erratum of relevant publication |
| Rolls B J. Effects of intense sweeteners on hunger, food intake, and body weight: a review. Am J Clin Nutr 1991;53(4):872-8. | Excluded study design |
| Rolls B J. Fat and sugar substitutes and the control of food intake. Ann N Y Acad Sci 1997;819:180-93. | Excluded study design |
| Romaguera D, Norat T, Wark P A, Vergnaud A C, Schulze M B, van Woudenbergh G J, Drogan D, Amiano P, Molina-Montes E, Sá¡nchez M J, Balkau B, Barricarte A, Beulens J W, Clavel-Chapelon F, Crispim S P, Fagherazzi G, Franks P W, Grote V A, Huybrechts I, Kaaks R, Key T J, Khaw K T, Nilsson P, Overvad K, Palli D, Panico S, Quirá³s J R, Rolandsson O, Sacerdote C, Sieri S, Slimani N, Spijkerman A M, Tjonneland A, Tormo M J, Tumino R, van den Berg S W, Wermeling P R, Zamara-Ros R, Feskens E J, Langenberg C, Sharp S J, Forouhi N G, Riboli E, Wareham N J. Consumption of sweet beverages and type 2 diabetes incidence in European adults: results from EPIC-InterAct. Diabetologia 2013;56(7):1520-30. | Excluded study design |
| Romo-Romo A, Aguilar-Salinas CA, Gómez-Díaz RA, Brito-Córdova GX, Gómez-Velasco DV, López-Rocha MJ, Almeda-Valdés P. Non-nutritive sweeteners: Evidence on their association with metabolic diseases and potential effects on glucose metabolism and appetite. Revista de Investigacion Clinica 2017;69(3):129-138. | Excluded study design |
| Rosenman K. Benefits of saccharin: a review. Environ Res 1978;15(1):70-81. | Excluded study design |
| Sánchez-Lozada LG, Le M, Segal M, Johnson RJ. How safe is fructose for persons with or without diabetes? American Journal of Clinical Nutrition 2008;88(5):1189-1190. | Excluded study design |
| Sardesai Vishwanath M, Waldshan Tammi H. Natural and synthetic intense sweeteners. The Journal of Nutritional Biochemistry 1991;2(5):236-244. | Excluded study design |
| Schillinger D, Kearns C. In response. Annals of Internal Medicine 2017;167(1):72-73. | Excluded study design |
| Schillinger D, Tran J, Mangurian C, Kearns C. Do sugar-sweetened beverages cause obesity and diabetes? Industry and the manufacture of scientific controversy. Annals of Internal Medicine 2016;165(12):895-897. | Excluded intervention/exposure |
| Schlienger J-L. The stormy history of sweet tastes: Between suspicions and realities. Medecine des Maladies Metaboliques 2020;14(2):126-135. | Publication not in English |
| Schneider BC, Dumith SC, Orlandi SP, Assunção MCF. Diet and body fat in adolescence and early adulthood: A systematic review of longitudinal studies. Ciencia e Saude Coletiva 2017;22(5):1539-1552. | Excluded intervention/exposure |
| Serra-Majem L, Raposo A, Aranceta-Bartrina J, Varela-Moreiras G, Logue C, Laviada H, Socolovsky S, Pérez-Rodrigo C, Aldrete-Velasco JA, Meneses Sierra E, López-García R, Ortiz-Andrellucchi A, Gómez-Candela C, Abreu R, Alexanderson E, Álvarez-Álvarez RJ, Álvarez Falcón AL, Anadón A, Bellisle F, Beristain-Navarrete IA, Blasco Redondo R, Bochicchio T, Camolas J, Cardini FG, Carocho M, Costa MDC, Drewnowski A, Durán S, Faundes V, Fernández-Condori R, García-Luna PP, Garnica JC, González-Gross M, La Vecchia C, Leis R, López-Sobaler AM, Madero MA, Marcos A, Mariscal Ramírez LA, Martyn DM, Mistura L, Moreno Rojas R, Moreno Villares JM, Niño-Cruz JA, Oliveira MBPP, Palacios Gil-Antuñano N, Pérez-Castells L, Ribas-Barba L, Rincón Pedrero R, Riobó P, Rivera Medina J, Tinoco de Faria C, Valdés-Ramos R, Vasco E, Wac SN, Wakida G, Wanden-Berghe C, Xóchihua Díaz L, Zúñiga-Guajardo S, Pyrogianni V, Cunha Velho de Sousa S. Ibero-American Consensus on Low- and No-Calorie Sweeteners: Safety, Nutritional Aspects and Benefits in Food and Beverages. Nutrients 2018;10(7):818. | Excluded study design |
| Shearer J, Swithers S E. Artificial sweeteners and metabolic dysregulation: Lessons learned from agriculture and the laboratory. Rev Endocr Metab Disord 2016;17(2):179-86. | Excluded study design |
| Sievenpiper J L. Low-carbohydrate diets and cardiometabolic health: the importance of carbohydrate quality over quantity. Nutr Rev 2020;78(Suppl 1):69-77. | Excluded study design |
| Sievenpiper JL, De Souza RJ. Are sugar-sweetened beverages the whole story? American Journal of Clinical Nutrition 2013;98(2):261-263. | Excluded study design |
| Sievenpiper JL. Fructose: Where does the truth lie? Journal of the American College of Nutrition 2012;31(3):149-151. | Excluded study design |
| Singh GM. Sugar sweetened beverages are associated with greater incidence of diabetes but there is a paucity of evidence on healthfulness of artificially-sweetened beverages and fruit juices. Evidence-Based Medicine 2016;21(1):35. | Excluded study design |
| Stanhope KL, Goran MI, Bosy-Westphal A, King JC, Schmidt LA, Schwarz J-M, Stice E, Sylvetsky AC, Turnbaugh PJ, Bray GA, Gardner CD, Havel PJ, Malik V, Mason AE, Ravussin E, Rosenbaum M, Welsh JA, Allister-Price C, Sigala DM, Greenwood MRC, Astrup A, Krauss RM. Pathways and mechanisms linking dietary components to cardiometabolic disease: thinking beyond calories. Obesity Reviews 2018;19(9):1205-1235. | Excluded study design |
| Stanhope KL. Sugar consumption, metabolic disease and obesity: The state of the controversy. Critical Reviews in Clinical Laboratory Sciences 2016;53(1):52-67. | Excluded study design |
| Stanley Lesley. Review of data on the food additive aspartame. EFSA SupportingPublications 2013;10(3):399E. | Excluded outcome |
| St-Onge M P, Heymsfield S B. Usefulness of artificial sweeteners for body weight control. Nutr Rev 2003;61(6 Pt 1):219-21. | Excluded study design |
| St-Onge M-P. Preventing insufficient sleep in adolescents: one step in helping them achieve a healthy lifestyle? Sleep 2022;45(5):zsac011. | Excluded study design |
| Sweetened drinks and increased incidence of type 2 diabetes? Drug and Therapeutics Bulletin 2015;53(10):112. | Excluded study design |
| Swithers S E. Artificial sweeteners are not the answer to childhood obesity. Appetite 2015;93:85-90. | Excluded study design |
| Swithers S E. Artificial sweeteners produce the counterintuitive effect of inducing metabolic derangements. Trends Endocrinol Metab 2013;24(9):431-41. | Excluded study design |
| Swithers SE. Not-so-healthy sugar substitutes? Current Opinion in Behavioral Sciences 2016;9:106-110. | Excluded study design |
| Sylvetsky A C, Rother K I. Nonnutritive Sweeteners in Weight Management and Chronic Disease: A Review. Obesity (Silver Spring) 2018;26(4):635-640. | Excluded study design |
| Sylvetsky A, Rother K I, Brown R. Artificial sweetener use among children: epidemiology, recommendations, metabolic outcomes, and future directions. Pediatr Clin North Am 2011;58(6):1467-80, xi. | Excluded study design |
| Te Morenga L, Mallard S, Mann J. Dietary sugars and body weight: systematic review and meta-analyses of randomised controlled trials and cohort studies. Bmj 2012;346:e7492. | Excluded intervention/exposure |
| The InterAct consortium. Consumption of sweet beverages and type 2 diabetes incidence in European adults: Results from EPIC-InterAct. Diabetologia 2013;56(7):1520-1530. | Excluded study design |
| The Lancet Diabetes & Endocrinology. Sweet success: will sugar taxes improve health? The Lancet Diabetes and Endocrinology 2017;5(4):235. | Excluded study design |
| Toi PL, Anothaisintawee T, Chaikledkaew U, Briones JR, Reutrakul S, Thakkinstian A. Preventive role of diet interventions and dietary factors in type 2 diabetes mellitus: An umbrella review. Nutrients 2020;12(9):44943. | Excluded outcome |
| Tucker R M, Tan S Y. Do non-nutritive sweeteners influence acute glucose homeostasis in humans? A systematic review. Physiol Behav 2017;182:17-26. | Excluded outcome |
| Valerino-Perea S, Lara-Castor L, Armstrong MEG, Papadaki A. Definition of the traditional mexican diet and its role in health: A systematic review. Nutrients 2019;11(11):2803. | Excluded intervention/exposure |
| von Philipsborn P, Stratil JM, Burns J, Busert LK, Pfadenhauer LM, Polus S, Holzapfel C, Hauner H, Rehfuess E. Environmental interventions to reduce the consumption of sugar-sweetened beverages and their effects on health. Cochrane Database of Systematic Reviews 2016;2016(7). | Excluded intervention/exposure |
| Warshaw H, Edelman SV. Practical strategies to help reduce added sugars consumption to support glycemic and weight management goals. Clinical Diabetes 2021;39(1):45-56. | Excluded study design |
| Yang Q. Gain weight by "going diet?" Artificial sweeteners and the neurobiology of sugar cravings: Neuroscience 2010. Yale J Biol Med 2010;83(2):101-8. | Excluded study design |
| Young J, Conway E M, Rother K I, Sylvetsky A C. Low-calorie sweetener use, weight, and metabolic health among children: A mini-review. Pediatr Obes 2019;14(8):e12521. | Excluded study design |
| Zdanowicz K, Białokoz-Kalinowska I, Lebensztejn DM. Non-alcoholic fatty liver disease in non-obese children. Hong Kong Medical Journal 2020;26(5):459-462. | Excluded study design |

## Supplemental Table 2. List of publications that met the a priori inclusion criteria but were not evaluated further in the current overview or reviews

| **Reference** | **Reason for exclusion** |
| --- | --- |
| Andrade L, Lee KM, Sylvetsky AC, Kirkpatrick SI. Low-calorie sweeteners and human health: A rapid review of systematic reviews. Nutrition Reviews 2021;79(10):1145-1164. | Overview of Reviews |
| Avery A, Bostock L, McCullough F. A systematic review investigating interventions that can help reduce consumption of sugar-sweetened beverages in children leading to changes in body fatness. J Hum Nutr Diet 2015;28 Suppl 1(Suppl 1):52-64. | Other intervention/exposure; LCS is the control |
| Daniels MC, Popkin BM. Impact of water intake on energy intake and weight status: A systematic review. Nutrition Reviews 2010;68(9):505-521. | Other intervention/exposure; LCS is the control |
| de la Hunty A, Gibson S, Ashwell M. A review of the effectiveness of aspartame in helping with weight control. Nutrition Bulletin 2006;31(2):115-128. | Non-systematic review with meta-analysis |
| Dennis E A, Flack K D, Davy B M. Beverage consumption and adult weight management: A review. Eat Behav 2009;10(4):237-46. | Systematic search with non-systematic narrative summary |
| Durán Agüero S, Angarita Dávila L, Escobar Contreras MC, Rojas Gómez D, De Assis Costa J. Noncaloric Sweeteners in Children: A Controversial Theme. BioMed Research International 2018. | Systematic search with non-systematic narrative summary |
| Fayet-Moore F. Effect of flavored milk vs plain milk on total milk intake and nutrient provision in children. Nutrition Reviews 2016;74(1):44943. | Other intervention/exposure; LCS is the control |
| Forshee RA, Anderson PA, Storey ML. Sugar-sweetened beverages and body mass index in children and adolescents: A meta-analysis. American Journal of Clinical Nutrition 2008;87(6):1662-1671. | Other intervention/exposure; LCS is the control |
| Gardner C, Wylie-Rosett J, Gidding S S, Steffen L M, Johnson R K, Reader D, Lichtenstein A H. Nonnutritive sweeteners: current use and health perspectives: a scientific statement from the American Heart Association and the American Diabetes Association. Circulation 2012;126(4):509-19. | Systematic search with non-systematic narrative summary |
| Higgins K A, Rawal R, Baer D J, O'Connor L E, Appleton K M. Scoping Review and Evidence Map on the Relationship Between Exposure to Dietary Sweetness and Body Weight-Related Outcoms In Adults. Adv Nutr 2022; 13(6):2341-2356. | Scoping review and/or evidence map |
| Hjelmesæth J, Sjöberg A. Human body weight, nutrients, and foods: a scoping review. Food and Nutrition Research 2022;66. | Scoping review of reviews |
| Hoare E, Varsamis P, Owen N, Dunstan DW, Jennings GL, Kingwell BA. Sugar-and intense-sweetened drinks in Australia: A systematic review on cardiometabolic risk. Nutrients 2017;9(10). | LCSB could not be isolated from SSB |
| Jakobsen DD, Brader L, Bruun JM. Effects of foods, beverages and macronutrients on BMI z-score and body composition in children and adolescents: a systematic review and meta-analysis of randomized controlled trials. European Journal of Nutrition 2022;62(1):1-15. | Other intervention/exposure; LCS is the control |
| Lam J, Elmore R, Howard B, Shah RR. Low-calorie sweeteners and health outcomes: an evaluation of rapid versus traditional evidence mapping. BMC Research Notes 2022;15(1). | Scoping review and/or evidence map |
| Lam J, Howard BE, Thayer K, Shah RR. Low-calorie sweeteners and health outcomes: A demonstration of rapid evidence mapping (rEM). Environment International 2019;123:451-458. | Scoping review and/or evidence map |
| Laviada-Molina HA, Molina-Seguí F, Arjona‐Villicana RD, Morales-Gual M, Cuello-García CA, Pérez-Gaxiola G. Nonâ€nutritive sweeteners for the prevention or treatment of being overweight or obesity. Cochrane Database of Systematic Reviews 2019;(4). | Withdrawn protocol |
| Lohner S, Toews I, Meerpohl JJ. Health outcomes of non-nutritive sweeteners: Analysis of the research landscape. Nutrition Journal 2017;16(1). | Scoping review and/or evidence map |
| Malik VS, Schulze MB, Hu FB. Intake of sugar-sweetened beverages and weight gain: A systematic review. American Journal of Clinical Nutrition 2006;84(2):274-288. | Other intervention/exposure; LCS is the control |
| Mandrioli D, Kearns CE, Bero LA. Relationship between research outcomes and risk of bias, study sponsorship, and author financial conflicts of interest in reviews of the effects of artificially sweetened beverages on weight outcomes: A systematic review of reviews. PLoS ONE 2016;11(9). | Overview of Reviews |
| Mosdøl A, Vist GE, Svendsen C, Dirven H, Laugsand Lillegaard IT, Mathisen GH, Husøy T. Hypotheses and evidence related to intense sweeteners and effects on appetite and body weight changes: A scoping review of reviews. PLoS ONE 2018;13(7). | Scoping review of reviews |
| Normand M, Ritz C, Mela D, Raben A. Low-energy sweeteners and body weight: A citation network analysis. BMJ Nutrition, Prevention and Health 2021;4(1):319-332. | Citation network analysis |
| Olivier B, Serge A H, Catherine A, Jacques B, Murielle B, Marie-Chantal C L, Sybil C, Jean-Philippe G, Sabine H, Esther K, Perrine N, Fabienne R, Gérard S, Irá¨ne M. Review of the nutritional benefits and risks related to intense sweeteners. Arch Public Health 2015;73:41. | Systematic search with non-systematic narrative summary |
| Patel AI, Moghadam SD, Freedman M, Hazari A, Fang M-L, Allen IE. The association of flavored milk consumption with milk and energy intake, and obesity: A systematic review. Preventive Medicine 2018;111:151-162. | Other intervention/exposure; LCS is the control |
| Pereira M A, Odegaard A O. Artificially sweetened beverages--do they influence cardiometabolic risk? Curr Atheroscler Rep 2013;15(12):375. | Systematic search with non-systematic narrative summary |
| Pereira M A. Diet beverages and the risk of obesity, diabetes, and cardiovascular disease: a review of the evidence. Nutr Rev 2013;71(7):433-40. | Systematic search with non-systematic narrative summary |
| Romo-Romo A, Aguilar-Salinas CA, Brito-Cordova GX, Diaz RAG, Valentin DV, Almeda-Valdes P. Effects of the non-nutritive sweeteners on glucose metabolism and appetite regulating hormones: Systematic review of observational prospective studies and clinical Trials. PLoS ONE 2016;11(8). | 1 study was included in the SR that evaluated obesity as an outcome; results related to obesity not summarized |
| Shankar P, Ahuja S, Sriram K. Non-nutritive sweeteners: Review and update. Nutrition 2013;29(45242):1293-1299. | Systematic search with non-systematic narrative summary |
| Vermunt S H, Pasman W J, Schaafsma G, Kardinaal A F. Effects of sugar intake on body weight: a review. Obes Rev 2003;4(2):91-9. | Systematic search with non-systematic narrative summary |
| Walbolt J, Koh Y. Non-nutritive sweeteners and their associations with obesity and type 2 diabetes. Journal of Obesity and Metabolic Syndrome 2020;29(2):114-123. | Systematic search with non-systematic narrative summary |
| Zheng M, Allman-Farinelli M, Heitmann BL, Rangan A. Substitution of Sugar-Sweetened Beverages with Other Beverage Alternatives: A Review of Long-Term Health Outcomes. Journal of the Academy of Nutrition and Dietetics 2015;115(5):767-779. | Other intervention/exposure; LCS is the control |

## *Supplemental Table 3. Certainty of evidence of the association between LCS intake and BW-related outcomes as reported in select systematic reviews*

| **Reference** | **Population** | **Study Design** | **Number of Studies** | **Intervention/ Exposure** | **Comparator** | **Outcome** | **Estimated Effect ^1^** | **Certainty of Evidence ^2^** |
| --- | --- | --- | --- | --- | --- | --- | --- | --- |
| **Any LCS** |  |  |  |  |  |  |  |  |
| Rios-Leyvraz 2022 | General adult population | RCT | 29 | Higher intake of LCS | Lower intake of LCS | BW | -0.71  (-1.13, -0.28) | Low |
| Rios-Leyvraz 2022 | General adult population | RCT | 23 | Higher intake of LCS | Lower intake of LCS | BMI | -0.14  (-0.30. 0.02) | Low |
| Rios-Leyvraz 2022 | General adult population | RCT | 4 | LCS | Sugars | BW | -0.61  (-1.28, 0.06) | Moderate |
| Rios-Leyvraz 2022 | General adult population | RCT | 4 | LCS | Sugars | BMI | -0.01  (-0.38, 0.35) | Moderate |
| Laviada-Molina 2020 | Total Population | RCT | 20 | LCS | All comparators | BW or BMI | SMD -0.40  (-0.57, -0.22) | Low |
| Laviada-Molina 2020 | Total Population | RCT | 13 | LCS | Sucrose | BW or BMI | SMD -0.56  (-0.79, -0.34) | Moderate |
| Laviada-Molina 2020 | Total Population | RCT | 4 | LCS | Water | BW or BMI | SMD -0.20  (-0.62, 0.23) | Low |
| Laviada-Molina 2020 | Total Population | RCT | 5 | LCS | Nothing or Placebo | BW or BMI | SMD -0.06  (-0.27, 0.15) | Moderate |
| Toews 2019 | Adults | RCT | 5 | LCS | Sugars or Placebo | BW | -1.29  (-2.80, 0.21) | Very low |
| Toews 2019 | Adults | RCT | 2 | LCS | Sugars | BMI | -0.60  (-1.19, -0.01) | Low |
| Rios-Leyvraz 2022 | General adult population | NRS (cont) | 4 | Higher intake of LCS (cont) | Lower intake of LCS (cont) | BW | -0.12  (-0.40, 0.15) | Very low |
| Rios-Leyvraz 2022 | General adult population | NRS (h v l) | 5 | Higher intake of LCS | Lower intake of LCS | BW | -0.01  (-0.67, 0.64) | Very low |
| Rios-Leyvraz 2022 | General adult population | NRS (h v l) | 5 | Higher intake of LCS | Lower intake of LCS | BMI | 0.14  (0.03, 0.25) | Very low |
| Rios-Leyvraz 2022 | General adult population | NRS | 2 | Higher intake of LCS | Lower intake of LCS | OB incidence | HR 1.76 (1.25, 2.49) | Low |
| **LNCSB** |  |  |  |  |  |  |  |  |
| McGlynn 2022 | Total Population | RCT | 24 (9) ^3^ | LCSB | Water | BW | -1.07  (-1.95, -0.19) | Low |
| McGlynn 2022 | Total Population | RCT | 14 (3) ^3^ | LCSB | Water | BMI | 0.02  (-0.46, 0.51) | Low |
| Lee 2022 | Total Population | Prospective cohort, substitution analysis | 1 | LCSB | Water | BW | MD 0.08  (-0.11, 0.27)  SMD 0.01 (-0.01, 0.02) | Very Low, Low^4^ |
| Lee 2022 | Total Population | Prospective cohort, substitution analysis | 1 | LCSB | Water | OB incidence | MD 1.19  (0.94, 1.50)  SMD 0.09 (-0.03, 0.22) | Very Low, Very Low^4^ |
| Lee 2022 | Total Population | Prospective cohort, change analysis | 5 | Change in LCSB (increasing 330 mL/day) | No Change in LCSB | BW | MD -0.008  (-0.014, -0.002)  SMD -0.01 (-0.02, -0.004) | Low, Low ^4^ |
| Toews 2019 | Adults | Prospective cohort | 1 | Higher LCS intake (>5.8 g saccharin) | Lower LCS intake (≤5.8 g saccharin) | BW | 0.09  (0.05, 0.13) | Very low |

BW, body weight; cont, continuous; LCS, low calorie sweetener; LCSB, low calorie sweetened beverage(s); NRS, non-randomized studies; RCT, randomized controlled trial; SMD, standardized mean difference; SSB, sugar sweetened beverage(s)

^1^ MD (95% CI) unless otherwise indicated.

^2^ GRADE unless otherwise indicated.

^3^ Number of studies included in the network meta-analysis (number of studies in direct meta-analysis).

^4^ GRADE and NutriGrade assessments, respectively.
